# Shielding of actin by the endoplasmic reticulum impacts nuclear positioning

**DOI:** 10.1038/s41467-022-30388-3

**Published:** 2022-05-19

**Authors:** Cátia Silva Janota, Andreia Pinto, Anna Pezzarossa, Pedro Machado, Judite Costa, Pedro Campinho, Cláudio A. Franco, Edgar R. Gomes

**Affiliations:** 1 Instituto de Medicina Molecular João Lobo Antunes, Faculdade de Medicina, Universidade de Lisboa, Lisboa, Portugal; 2 Electron Microscopy Core Facility (EMCF), European Molecular Biology Laboratory, Heidelberg, Germany; 3 Instituto de Histologia e Biologia do Desenvolvimento, Faculdade de Medicina, Universidade de Lisboa, Lisboa, Portugal; 4 Present Address: Royal Brompton Hospital and Harefield NHS Foundation Trust, London, UK; 5 Present Address: Champalimaud Foundation, Champalimaud Centre for the Unknown, Lisbon, Portugal; 6 Present Address: Centre for Ultrastructural Imaging, King’s College London, London, UK

**Keywords:** Endoplasmic reticulum, Mesenchymal migration

## Abstract

Nuclear position is central to cell polarization, and its disruption is associated with various pathologies. The nucleus is moved away from the leading edge of migrating cells through its connection to moving dorsal actin cables, and the absence of connections to immobile ventral stress fibers. It is unclear how these asymmetric nucleo-cytoskeleton connections are established. Here, using an in vitro wound assay, we find that remodeling of endoplasmic reticulum (ER) impacts nuclear positioning through the formation of a barrier that shields immobile ventral stress fibers. The remodeling of ER and perinuclear ER accumulation is mediated by the ER shaping protein Climp-63. Furthermore, ectopic recruitment of the ER to stress fibers restores nuclear positioning in the absence of Climp-63. Our findings suggest that the ER mediates asymmetric nucleo-cytoskeleton connections to position the nucleus.

## Abstract (web summary)

The nucleus connects to the actin cytoskeleton for nuclear movement in migrating cells. Here, the authors show that the endoplasmic reticulum shields actin cables to generate asymmetric nucleo-cytoskeleton connections for nuclear positioning.

## Introduction

The cell nucleus is often depicted in textbooks as floating in the center of the cell. However, the position of the nucleus is highly regulated, and it is associated with cell function^1^. The position of the nucleus often changes during different biological processes, such as cell division, cell differentiation, and cell migration^2–4^. Mounting evidences suggest that nuclei positioning is associated with specialized cellular functions, and that misregulation of nuclear positioning could lead to cell dysfunction and diseases, such as centronuclear myopathies, progeria, and lissencephaly^5,6^.

Nuclear positioning often requires connections of the nucleus with the cytoskeleton^7^. These nucleo-cytoskeleton connections are mostly meditated by the LInker of Nucleoskeleton and Cytoskeleton (LINC) complex at the nuclear envelope^8^. The LINC complex is composed of outer nuclear envelope (ONM) proteins of the Nesprin family that interact with the cytoskeleton, and inner nuclear envelope (INM) proteins of the SUN family, that interact with the nuclear lamina. The KASH domains of Nesprin proteins interact with the SUN domains of SUN proteins in the nuclear envelope lumen^9^. Multiple proteins have been found to be involved in nucleo-cytoskeleton connections by the LINC complex, however it is unknown how these nucleo-cytoskeleton connections are turned on and off during nuclear positioning^10,11^.

The position of the nucleus during cell migration is of most importance, since it can affect the speed of migration^10,12^, the selection of the path of least resistance^13^, and the breaching through endothelial barriers^14^. The wound healing assay is a classic approach to study cell migration where wound-edge cells are stimulated with serum factor lysophosphatidic acid (LPA) to move their nucleus away from the leading edge^12,15^. The rearward movement of the nucleus is driven by moving dorsal actin cables coupled to the nucleus by linear arrays of the nuclear envelope protein Nesprin-2G, known as transmembrane actin-associated (TAN) nuclear lines^12,15^. During nuclear movement, the nucleus does not connect to the immobile ventral stress fibers, although Nesprin-2G is symmetrically distributed throughout the nuclear envelope^12^. Thus, the nucleus is asymmetrically connected to dorsal actin cables but not to ventral stress fibers during nuclear movement. It is unknown how these asymmetric nucleo-cytoskeleton connections are established.

The endoplasmic reticulum (ER) is contiguous with the nuclear envelope, and it is the central node in the organelle interactome network^16^. The ER spreads throughout the cytoplasm as an interconnected complex network of flat structures (sheets), reticular networks (tubules), and dense meshwork of tubules (ER matrices). The ER complex morphology and distribution is regulated by ER-shaping proteins^17–19^. Therefore, we sought to investigate if ER morphology and distribution could regulate nuclear positioning. Here we reveal that the ER is involved in the establishment of asymmetric nucleo-cytoskeleton connections required for nuclear positioning.

## Results

### Nuclear positioning is associated with ER morphology remodeling and requires Climp-63

**Fig. 1:**
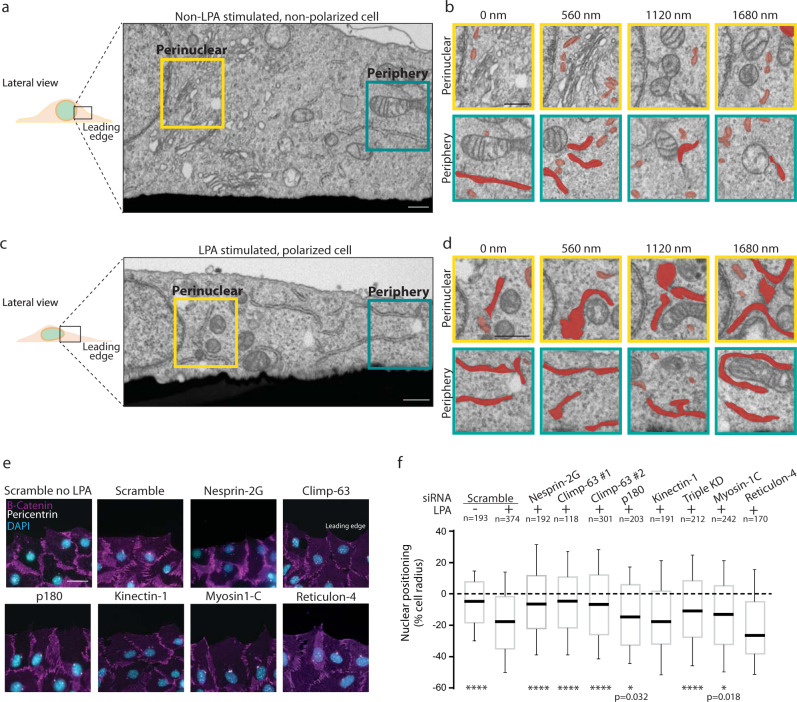
Nuclear positioning is associated with ER morphology remodeling and requires Climp-63. **a** FIB-SEM representative image of non-LPA stimulated, non-polarized wound-edge U2OS cell. One stack of 998 SEM images from one leading edge cell. **b** Representative images of perinuclear (yellow insets) and peripheral (green insets) regions. The ER is highlighted in red, light red represents tubules (<1 µm), dark red represents sheets (>1 µm). Measurements represent the depth of the section. **c** as in **a**, but an LPA stimulated and polarized cell. One stack of 1798 SEM images from one leading edge cell. **d** as in **b**, but an LPA stimulated and polarized cell. **e** Representative widefield epifluorescence images of wound-edge NIH3T3 fibroblasts. Cells were immunostained for cell-cell contacts (purple; β-catenin), centrosome (white; pericentrin) and DNA (DAPI, blue). **f** Nuclear positions relative to the cell centroid of cells represented in **e**. Significance (Two-tailed unpaired t-test) was calculated between experimental condition and the scramble siRNA with LPA. ****p < 0.0001 (Scramble no LPA, Nesprin-2G, Climp-63 #1, Climp-63 #2, Triple KD); *p < 0.05. Box shows first quartile, median and third quartile, and whiskers show 10–90% percentile. Experiments were repeated ≥3, except (**a**–**d**). Scale bars: **a**, **b**, **c**, **d** 1 µm; **e** 40 µm.

It is not known how the ER organizes during nuclear positioning. Therefore, we investigated the ER morphology and distribution during nuclear positioning in migrating NIH3T3 fibroblasts. We used the wound healing assay, where LPA induces rearward nuclear positioning in wound-edge cells. To understand how the ER organizes during nuclear positioning, we analyzed the ER in non-LPA cells (non-polarized) and LPA stimulated cells (polarized) at the wound edge by using focused ion beam scanning electron microscopy (FIB-SEM) (Fig. 1a–d). The ER can form sheets, tubules or matrices mediated by ER-shaping proteins^17–19^. Analysis of FIB-SEM cellular volume revealed a substantial difference in ER morphology and distribution between non-LPA and LPA stimulated cells. The LPA stimulated cell presented an increase in ER sheets (dark red), at the expense of a reduction in ER tubules (light red), when compared to the non-LPA stimulated cell (Fig. 1a–d). Additionally, the LPA stimulated cell exhibited an accumulation of perinuclear ER, when compared to the non-LPA stimulated cell (Fig. 1b, d; Supplementary Fig. 1a, b). The acquisition conditions used for FIB-SEM were selected to result in 8 nm step in the Z position, which allowed us to segment and render in three dimensional (3D) the remarkably complex and convoluted ER structures. The analysis of ER morphology in 3D confirmed that perinuclear ER is mostly organized in sheets in the LPA stimulated cell, whereas non-LPA stimulated cell presented more ER tubules (Supplementary Fig. 1a). These results suggest that during nuclear positioning ER accumulates at the perinuclear region and undergoes a tubules-to-sheets transition.

To test whether ER morphology is involved in rearward nuclear positioning, we manipulated several proteins that modulate ER sheets-to-tubules ratio and ER distribution, known as ER-shaping proteins^20^. We depleted ER-sheets related proteins (Climp-63, p180, Kinectin-1, Myosin-1C), and a protein involved in ER tubules maintenance (Reticulon-4) (Fig. 1e, f; Supplementary Fig. 1c, d)^17–19,21–24^. Depletion of Climp-63 (cytoskeleton-linking membrane protein 63) blocked rearward nuclear positioning in wound-edge fibroblasts upon LPA stimulation to the same degree as in Nesprin-2G depleted cells (Fig. 1f). Depletion of other proteins involved in ER sheet morphology (p180, Kinectin-1, and Myosin-1C) also blocked nuclear positioning, but only by 40% when compared to Nesprin-2G depleted cells. Depletion of Reticulon-4 (associated with ER tubule formation) did not affect rearward nuclear positioning. To further study how changes in ER morphology and distribution blocked nuclear movement, we performed time-lapse microscopy during LPA-stimulated nuclear movement in scramble and Climp-63 siRNA cells (Supplementary Fig. 1e). Quantification of persistence and directional persistence of nuclear movement revealed that Climp-63 depletion impaired rearward nuclear movement, when compared to scramble siRNA leading-edge cells (Supplementary Fig. 1f–h). Thus, the ER-shaping protein Climp-63 is required for nuclear positioning.

### ER accumulates at the perinuclear region during nuclear positioning and requires Climp-63

**Fig. 2:**
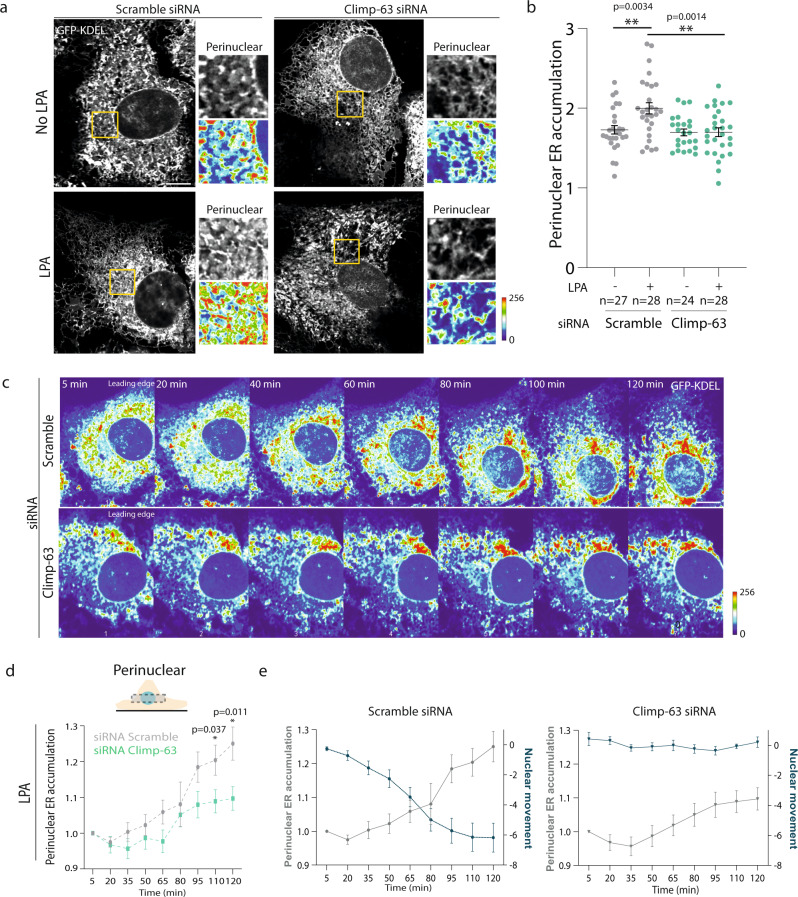
ER accumulates at the perinuclear region during nuclear positioning and requires Climp-63. **a** Representative point-scanning confocal Airyscan images of wound-edge GFP-KDEL-expressing fibroblasts treated with scramble or Climp-63 siRNA, stimulated and non-stimulated with LPA. Perinuclear region of interest (ROI) are highlighted (yellow box) and represented as grey scale (top) and as heatmap (bottom). **b** Quantification of perinuclear ER accumulation of cells represented in **a**. treated with scramble (grey) or Climp-63 (green) siRNA. Significance (Two-tailed unpaired *t*-test) was calculated between experimental conditions and scramble with LPA (second condition). **c** Fluorescence spinning-disk confocal kymograph of nuclear movement and perinuclear ER accumulation in wound-edge GFP-KDEL-expressing cells treated with scramble (top) or Climp-63 (bottom) siRNA upon LPA stimulation. Images correspond to the maximal intensity projection of z-stacks from ventral to dorsal side of the cell. The signal intensity is represented as a thermal heatmap. **d** Quantification of perinuclear ER accumulation in LPA stimulated cells treated with scramble (grey, *n* = 22 cells of 4 independent experiments) or Climp-63 (green, *n* = 21 cells of 4 independent experiments) siRNA, over time, represented in **c**. Significance (Two-tailed unpaired *t*-test) was calculated between scramble and Climp-63 siRNAs for each time point. **e** Quantification of perinuclear ER accumulation (grey line) and the nuclear movement over time (blue line), in LPA stimulated cells in scramble (left, *n* = 61 cells of 4 independent experiments) or Climp-63 (right, *n* = 37 cells of 4 independent experiments) siRNA. Perinuclear ER accumulation is the same data as in **d**. Scale bars: **a**, **c** 10 µm. Error bars, SEM. **p < 0.01, *p < 0.05.

To investigate whether Climp-63 has a role in perinuclear ER accumulation during nuclear positioning we analyzed the ER in wound-edge non-LPA and LPA stimulated fibroblasts. In non-LPA stimulated cells, Climp-63 depletion did not interfere with perinuclear ER accumulation (Fig. 2a, b). However, upon LPA stimulation we observed an increase of perinuclear ER accumulation that was dependent on Climp-63 (Fig. 2a, b).

Next, time-lapse microscopy of wound-edge scramble siRNA cells stably transfected with GFP-KDEL, which marks the ER, was used to investigate the relationship between nuclear positioning and perinuclear ER compaction upon LPA stimulation In scramble siRNA cells, LPA stimulation led to ER accumulation at the perinuclear region that correlated with rearward nuclear movement (Fig. 2c–e (left); Supplementary Movie 1). In the absence of Climp-63 we observed a minor accumulation of perinuclear ER upon LPA stimulation while no reward nuclear movement was observed (Fig. 2c–e (right), Supplementary Movie 2). Scramble siRNA cells increased in 25% the perinuclear ER, 120 min upon LPA stimulation when compared to 5 min. On the other hand, Climp-63 siRNA knockdown (KD) cells only increased 9% (120 min vs 5 min) (Fig. 2d). In the absence of LPA, cells treated with Scramble or Climp-63 siRNA did not exhibit perinuclear ER compaction nor nuclear movement over time (Supplementary Fig. 2a, b). These results indicate that perinuclear ER accumulation occurs in a Climp-63-dependent manner during nuclear positioning.

### Perinuclear ER accumulation depends on Climp-63 luminal domain but it is independent of microtubules

**Fig. 3:**
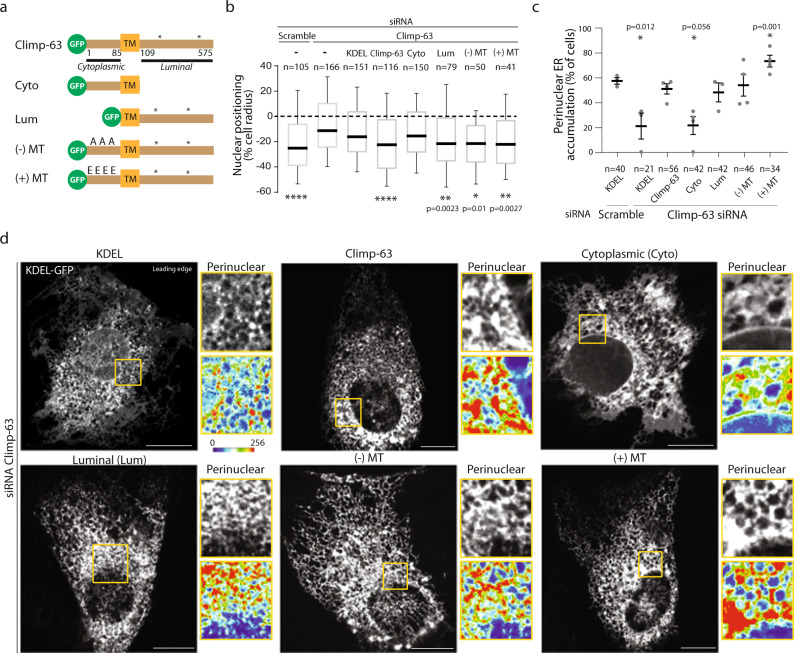
Perinuclear ER accumulation depends on Climp-63 luminal domain but it is independent of microtubules. **a** Schematic representation of Climp-63 synthetized plasmids. Climp-63 is the full-length protein. The cytoplasmic (Cyto) mutant lacks the luminal domain. The luminal (Lum) mutant lacks the cytoplasmic domain. The (−) MT mutant does not bind to microtubules. The (+) MT mutant is constitutively bound to microtubules. The asterisks represent the siRNA resistant mutations. **b** Average nuclear positions relative to the cell centroid of cells treated with scramble or Climp-63 siRNA and microinjected with the indicated plasmids. Significance (Two-tailed unpaired *t*-test) was calculated between the experimental conditions and Climp-63 siRNA cells (second condition). ****p < 0.0001 (Scramble alone, Climp-63); Box show first quartile, median and third quartile, and whiskers show 10–90% percentile. **c** Quantification of the percentage of cells with perinuclear ER accumulation. Significance (Two-tailed unpaired *t*-test) was calculated between experimental conditions and Climp-63 expressing cells (third condition). Error bars, SEM. **d** Representative point-scanning confocal images of wound-edge cells treated with Climp-63 siRNA and microinjected with the plasmids shown in **a**. Perinuclear ROI are highlighted (yellow box) and represented as grey scale (top) and as heatmap (bottom). Experiments were repeated ≥3. Scale bars: **d** 10 µm. **p < 0.01, *p < 0.05.

Climp-63 is an integral ER single-spanning transmembrane protein with a luminal and a cytoplasmic domain^25,26^ (Fig. 3a). The homo-dimerization of its luminal domain is involved in ER luminal width regulation and it makes Climp-63 a ER luminal spacer^19,21^. On the other hand, its cytoplasmic domain binds to microtubules in a phosphorylation-dependent manner, which contributes to ER sheet distribution^19,21,27^. Climp-63 is mainly associated with perinuclear ER sheet maintenance^21^. To identify the domains of Climp-63 involved in nuclear positioning and perinuclear ER accumulation, we microinjected a series of Climp-63 fragments into wound-edge Climp-63 depleted fibroblasts (Fig. 3a). The expression of the luminal domain, but not the cytoplasmic domain, in Climp-63 depleted cells was sufficient to restore nuclear positioning and perinuclear ER accumulation (Fig. 3b–d). Expression of full length Climp-63 constructs with mutations in the microtubule-binding domain that prevent or increase the interaction with microtubules was sufficient to restore not only nuclear positioning, but also perinuclear ER accumulation (Fig. 3b–d)^26,28^. Moreover, Climp-63 depletion does not affect the microtubule network in wound-edge cells (Supplementary Fig. 2c). These results demonstrate that Climp-63 luminal domain is sufficient to rescue nuclear positioning and perinuclear ER accumulation, independently of Climp-63 interaction with microtubules. Thus, we hypothesize that the perinuclear ER accumulation is required for nuclear positioning, and that changes in ER morphology may also be involved in the process.

### ER accumulates at the ventral and dorsal nuclear region without interfering with nuclear envelope TAN lines

**Fig. 4:**
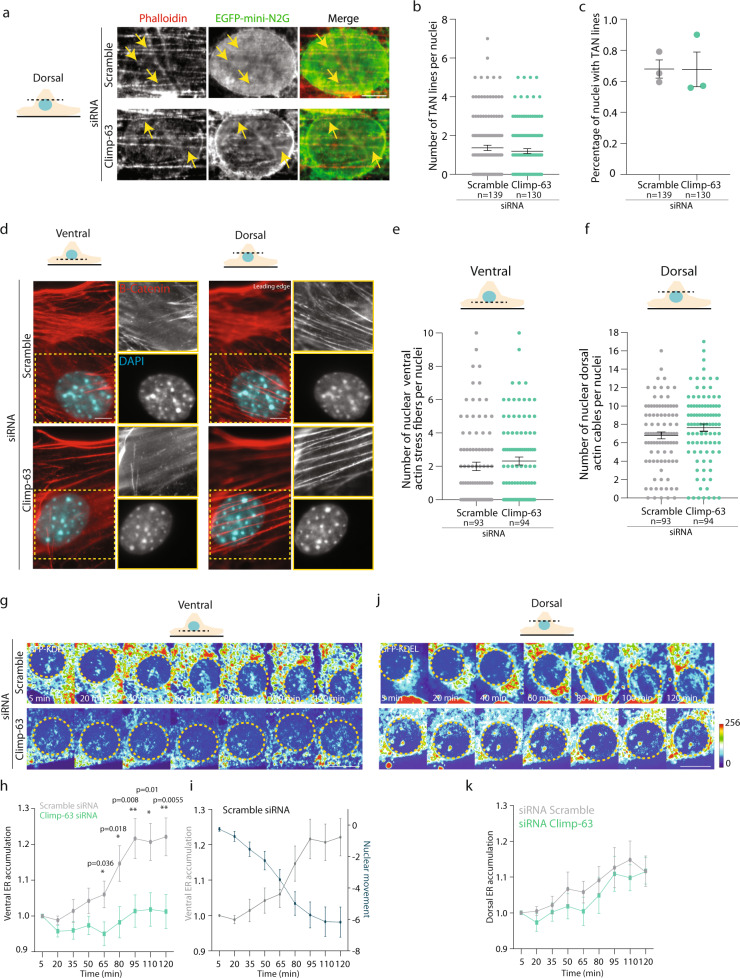
ER accumulates at the ventral and dorsal nuclear region during nuclear positioning without interfering with the connection of dorsal actin with nuclear envelope TAN lines. **a** Representative epifluorescence images of scramble or Climp-63 siRNA treated fibroblasts expressing GFP-mini-N2G and stained with phalloidin (F-actin). Arrows show colocalization of Nesprin-2G with dorsal actin cables. **b** Quantification of number of TAN lines per nuclei in cells treated with scramble (grey) or Climp-63 (green) siRNA as in **a**. **c** Quantification of the percentage of nuclei with TAN lines in cells treated with scramble (grey) or Climp-63 (green) siRNA as in **a**. **d** Representative epifluorescence images of wound-edge cells after LPA stimulation. Cells were stained as follows: F-actin (Phalloidin, red), DNA (DAPI, blue). Nuclear region highlighted with a yellow dashed box. Left: Ventral focal plane. Right: Dorsal focal plane. **e** Quantification of the number of nuclear ventral stress fibers per nuclei in cells treated with scramble (grey) or Climp-63 (green) siRNA as in **d**. **f** Quantification of the number of nuclear dorsal actin cables per nuclei in cells treated with scramble (grey) or Climp-63 (green) siRNA as in **d**. **g** Fluorescence kymograph of nuclear movement and ventral ER accumulation in wound-edge GFP-KDEL-expressing cells treated with scramble (top) or Climp-63 (bottom) siRNA upon LPA stimulation. **h** Quantification of ventral ER accumulation during LPA stimulation in scramble (grey) or Climp-63 (green) siRNA treated cells, represented in **g**. Significance (Two-tailed unpaired t-test) was calculated between scramble and Climp-63 siRNAs for each time point. **i** Quantification of perinuclear ER accumulation (grey line) and the nuclear movement over time (blue line), during LPA stimulation of cells represented in **g**. The grey line corresponds to the grey line in **h**. **j** Fluorescence kymograph of nuclear movement and dorsal ER accumulation in wound-edge GFP-KDEL-expressing cells treated with scramble (top) or Climp-63 (bottom) siRNA upon LPA stimulation. **k** Quantification of dorsal ER accumulation during LPA stimulation in scramble (grey) or Climp-63 (green) siRNA treated cells, represented in **j**. Experiments were repeated 3 times. Scale bars: **a**, **d** 5 µm; **g**, **j** 20 µm. Error bars, SEM. **p < 0.01, *p < 0.05.

Nuclear positioning in wound-edge fibroblasts is driven by actin retrograde flow, which moves dorsal actin cables away from the leading edge. When these dorsal cables reach the dorsal side of the nucleus, they anchor to Nesprin-2G at the nuclear envelope and trigger the formation of linear arrays of Nesprin-2G, known as TAN lines^12,29–31^. Thus, the movement of dorsal actin cables anchored to TAN lines drive nuclear movement. We analyzed the role of Climp-63 on actin retrograde flow, TAN lines and actin organization. To measure actin retrograde flow, we used stably transfected cells expressing LifeAct-mCherry and found that Climp-63 depletion did not interfere with actin retrograde flow directionality and speed, when compared to scramble siRNA cells (Supplementary Fig. 3a–c). Expression of Climp-63 luminal domain in Climp-63 siRNA cells (that rescues perinuclear ER accumulation and nuclear positioning) also did not affect actin retrograde flow speed, when compared to scramble siRNA cells (Supplementary Fig. 3c). To investigate TAN lines assembly, we microinjected scramble and Climp-63 siRNA treated wound-edge cells with EGFP-mini-N2G, a probe that accumulates at TAN lines^12^ and found that Climp-63 depletion does not affect the number of TAN lines per nucleus, nor the percentage of nuclei with TAN lines (Fig. 4a–c). Moreover, analysis of actin cytoskeleton organization revealed that Climp-63 KD did not affect actin network architecture at the perinuclear region. We did not observe any changes on the morphology and number of ventral stress fibers or dorsal actin cables per nuclei, between scramble and Climp-63 siRNA treated cells (Fig. 4d–f). Therefore, these results indicate that Climp-63 and perinuclear ER accumulation are not involved in the engagement of actin retrograde flow and on the connection of dorsal actin with nuclear envelope TAN lines on the dorsal side of the nucleus.

Next, we investigated the accumulation of ER on the dorsal and ventral side of the nucleus during nuclear movement. We quantified the perinuclear accumulation of the ER marker KDEL during nuclear positioning upon LPA stimulation at the dorsal and ventral side of the nucleus in scramble and Climp-63 siRNA treated cells (Fig. 4g–k). On the ventral side of the nucleus, we observed a significant increase of ER accumulation during nuclear movement in scramble siRNA cells, whereas no increase was observed in Climp-63 siRNA cells (Fig. 4g–h). Furthermore, nuclear movement correlates with ventral ER accumulation (Fig. 4i). In contrast, we observed no difference of dorsal ER accumulation between scramble and Climp-63 siRNA treated cells during nuclear movement (Fig. 4j, k). The different role of Climp-63 on the accumulation of the ER in the ventral versus dorsal side of the nucleus was not due to Climp-63 localization since microinjected GFP-Climp-63 in Climp-63 depleted cells was equally distributed in the ventral and dorsal side of the nucleus (Supplementary Fig. 4a, b).

Super resolution microcopy techniques, Airyscan and Structure illumination microscopy (SIM) further validated the reduction of ER in the ventral region of the nucleus in Climp-63 KD cells, when compared to scramble KO cells (Supplementary Fig. 4c–e). Additionally, transmission electron microscopy (TEM) of sagittal sections of wound-edge cells revealed a reduction of ventral ER luminal width and area upon Climp-63 depletion, whereas nuclear envelope luminal width was not affected (Supplementary Fig. 4f–j). The reduction of ER lumen width upon Climp-63 depletion was similar to the previously reported reduction of non-ventral ER lumen in Climp-63 depleted cells^19,21^. These results indicate that the ER accumulates both on the ventral and dorsal regions during nuclear positioning. The accumulation of the ER in the ventral side is Climp-63 dependent. On the other hand, the accumulation of ER in the dorsal side is independent of Climp-63 and does not interfere with anchoring of the nucleus to dorsal actin cables that drive nuclear movement.

### Ventral actin is wrapped up by ER during nuclear positioning

During nuclear positioning, the nucleus establishes asymmetric nucleo-cytoskeleton connections by binding to moving dorsal actin, but not to immobile ventral stress fibers^12^. While dorsal actin is engaged in actin retrograde flow, ventral stress fibers connect to focal adhesions and are therefore immobile. It remains unclear why ventral stress fibers do not connect to the nucleus, even though Nesprin-2G and other LINC Complex proteins are symmetrically distributed throughout the nuclear envelope^10,12^.

**Fig. 5:**
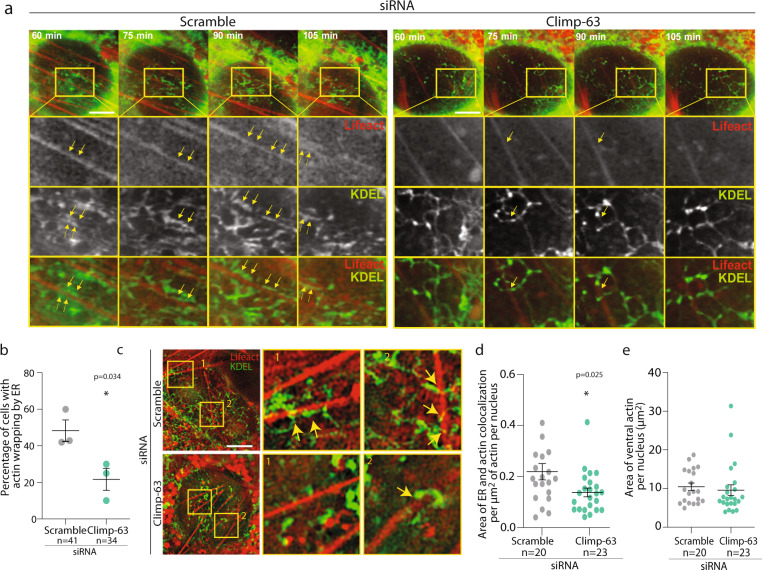
Ventral actin is wrapped up by ER during nuclear positioning. **a** Representative structure illumination microscopy (SIM) images of a time-lapse video during nuclear movement of wound-edge GFP-KDEL and LifeAct-mCherry fibroblasts treated with scramble and Climp-63 siRNAs. Arrows in highlighted ROI show ventral actin shielding by ER during nuclear movement. **b** Quantification of the percentage of cells with ER shielding of actin ventral stress fibers in cells treated with scramble (grey) or Climp-63 (green) siRNA as in **a**. **c** Representative structure illumination microscopy (SIM) images of wound-edge GFP-KDEL and LifeAct-mCherry cells treated with scramble (grey) and Climp-63 (green) siRNAs. ROI are highlighted (yellow box) and represented as x3 magnifications (right). **d** Quantification of area of ER and actin colocalization per um^2^ of actin per nucleus in cells treated with scramble (grey) or Climp-63 (green) siRNA as in **c**. **e** Quantification of nuclear ventral actin area in cells treated with scramble (grey) or Climp-63 (green) siRNA as in **c**. Significance (Two-tailed unpaired *t*-test) was calculated between scramble and Climp-63 siRNA. Experiments were repeated ≥3. Scale bars: **a**, **c** 5 µm. Error bars, SEM. *p < 0.05.

To understand whether the accumulation of ventral ER during nuclear positioning was associated with ventral stress fibers we performed time-lapse SIM of actin and ER on the ventral side of the nucleus in wound-edge cells during nuclear positioning. We observed that the ventral ER wraps around and encloses the ventral stress fibers (Fig. 5a), consistent with our previous observation that during nuclear positioning ER accumulates at the perinuclear region. The ventral ER exhibited minor displacements along these ventral stress fibers during nuclear movement suggesting a close and dynamic interaction between the ER and ventral stress fibers. Z-stacking imaging revealed two main types of interactions, the ER wrapped actin along the stress fiber, or resembled hooks of ER around fibers (Supplementary Fig. 5a, Supplementary Movie 3–4). In cells depleted for Climp-63, we observed a reduction in the number of cells with ventral stress fibers wrapped by ER during nuclear positioning (Fig. 5a, b). To quantify the wrapping of actin by ER, we measured the area of colocalization of ER (GFP-KDEL) with ventral stress fibers (LifeAct-mCherry) using SIM. The area of ER and actin colocalization was also reduced in Climp-63 KD cells when compared to scramble siRNA cells (Fig. 5c, d). The area of ventral actin was not different between scramble and Climp-63 siRNA cells (Fig. 5c, e). Super resolution imaging of the dorsal nuclear region showed no differences in area of dorsal ER and actin coverage by ER between Scramble and Climp-63 siRNA cells (Supplementary Fig. 5b–d). This observation is consistent with our observations that Climp-63 is not involved in accumulation of dorsal ER, nor on the establishment of TAN lines (Fig. 4). Moreover, depletion of Climp-63 did not affect Nesprin-2G distribution in the nuclear envelope, nor nuclear shape (Supplementary Fig. 6a–e). Furthermore, ER amount was not affected by depletion of Climp-63, as previously reported (Supplementary Fig. 6f)^21^. Based on these results, we hypothesized that wrapping of actin stress fibers by the ER could act as a shield preventing anchoring of the nucleus to the immobile ventral stress fibers. Thus, in the absence of Climp-63, when ER wrapping of actin stress fibers fails to occur, the nucleus would become anchored to the immobile ventral stress fibers and it would be unable to move.

### Recruitment of ER to ventral actin restores nuclear positioning

**Fig. 6:**
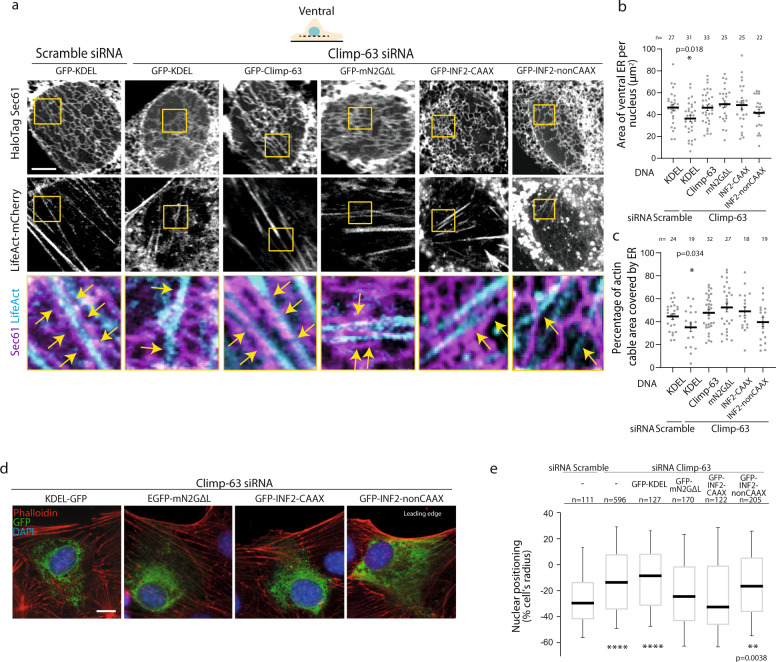
Recruitment of ER to ventral actin restores nuclear positioning. **a** Representative confocal Airyscan images of wound-edge cells stably expressing LifeAct-mCherry, and microinjected with HaloTag-Sec61 together with GFP-KDEL, GFP-Climp-63 or GFP-mN2GΔL, GFP-INF2-CAAX and GFP-INF2-nonCAAX. The highlighted ROI (yellow box) represent the insets presented below. **b** Quantification of area of ventral ER in cells treated as in **a**. Error bars, SEM. Significance (Two-tailed unpaired *t*-test) was calculated between experimental conditions and the first condition. **c** Quantification of percentage of actin cable area covered by ER in cells treated as in **a**. Error bars, SEM. Significance ((Two-tailed unpaired *t*-test) was calculated between experimental conditions and the first condition. **d** Epifluorescence representative images of Climp-63 siRNA treated wound-edge cells microinjected with GFP-KDEL, GFP-mN2GΔL GFP-INF2-CAAX or GFP-INF2-nonCAAX. Cells were stained as follows: actin (phalloidin, red), GFP (green), DNA (DAPI, blue). **e** Nuclear positions relative to the cell centroid in cells treated as in **d**. Box show first quartile, median and third quartile, and whiskers show 10–90% percentile. Significance (Two-tailed unpaired *t*-test) was calculated between experimental condition and the scramble siRNA. ****p < 0.0001 (siRNA Climp-63 alone, GFP-KDEL), **p < 0.01, *p < 0.05. Experiments were repeated ≥3. Scale bar: **a** 5 µm, **d** 10 µm.

To test if wrapping of actin by the ER is required for nuclear positioning, we devised two strategies to recruit ER to actin fibers in Climp-63 KD cells. It was previously shown that GFP-mini-N2G lacking the luminal region (GFP-m-N2GΔL) does not interact with the LINC complex, and therefore it is distributed throughout the ER. GFP-m-N2GΔL can bind to actin via the N-terminal CH domain^12^. Thus, GFP-m-N2GΔL can recruit ER to actin cytoskeleton. We found that expression by microinjection of GFP-m-N2GΔL in Climp-63 KD wound-edge cells restored ventral ER area and actin stress fibers wrapping by the ER to the same level as cells microinjected with Climp-63, and scramble siRNA treated cells (Fig. 6a–c). On the dorsal side, no differences were observed on ER area, dorsal actin wrapping by ER and TAN lines formation upon expression of GFP-mN2GΔL in Climp-63 siRNA cells, when compared to Scramble siRNA cells (Supplementary Fig. 5b–d, Supplementary Fig. 6g, h). Importantly, expression of GFP-m-N2GΔL also rescued nuclear positioning to the same level as cells microinjected with Climp-63, and scramble siRNA treated cell (Fig. 6d, e).

The second strategy we used to recruit ER to actin fibers involved Inverted Formin-2 (INF2). INF2 has two splicing isoforms, CAAX with localizes to the ER, and non-CAAX which localizes to focal adhesions and cytoplasm^32,33^. Previous results have shown that expression of GFP-INF2-CAAX, but not INF2 without the CAAX domain (GFP-INF2-nonCAAX), induces actin enrichment around the ER^34^. In line with these previous results, we found that expression of GFP-INF2-CAAX in Climp-63 siRNA cells restored ventral ER area, actin stress fibers wrapping by the ER, and nuclear positioning to the same level as cells microinjected with Climp-63, and scramble siRNA treated cells (Fig. 6a–e). On the other hand, overexpression of INF2 without the CAAX domain (GFP-INF2-noCAAX) in Climp-63 siRNA cells did not rescue nuclear positioning, and partially restored ventral ER area, and actin stress fibers wrapping by the ER (Fig. 6a–e). On the dorsal side, no differences were observed on ER area, dorsal actin wrapping by ER and TAN lines formation upon expression of INF2 isoforms in Climp-63 siRNA cells, when compared to Scramble siRNA cells (Supplementary Fig. 5b–d, Supplementary Fig. 6g, h). Thus, these results indicate that the recruitment of ER to ventral actin is sufficient to rescue nuclear positioning in the absence of Climp-63, likely through inhibition of ventral nucleo-cytoskeleton connections.

## Discussion

The position of the nucleus is an hallmark of cell polarization, and most nuclear positioning mechanisms are based on nucleo-cytoskeleton interactions^5,35–40^. Here we describe how the ER can generates asymmetric nucleo-cytoskeleton connections required for nuclear movement.

**Fig. 7:**
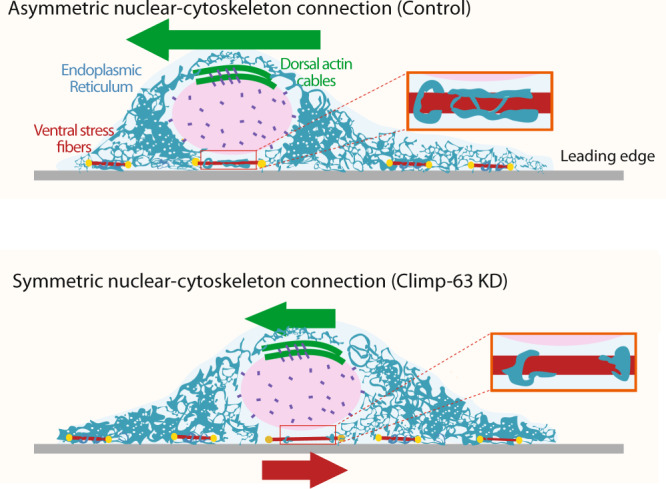
Working model. Diagram of sagittal view of two wound–edge cells, with the leading edge on the right side, on a substrate (light grey). Top Cell: Upon LPA stimulation (control), the nucleus moves away from the leading edge, while the ER accumulates at the perinuclear region. As a result, the ventral ER shields immobile ventral stress fibers and the nucleus moves away from the leading edge connected to dorsal actin cables. Bottom cell: In the absence of Climp-63, there is a reduction of perinuclear ER accumulation leading to a reduction of ventral stress fibers shielding by ER. Thus, the nucleus may interact with immobile ventral stress fibers resulting in a tug-of-war between dorsal actin cables trying to move the nucleus rearwards, and ventral stress fibers anchoring the nucleus.

We found that Climp-63 is required for nuclear positioning and ventral, but not dorsal, ER accumulation. Recruitment of ER to actin restores nuclear movement in Climp-63 depleted cells. Our results suggest that in the absence of Climp-63, the immobile ventral stress fibers are not wrapped by ER and thus can interact with the nucleus by an unidentified mechanism, thus preventing nuclear movement by dorsal actin cables. Therefore, the ER can act as a shield for ventral nucleo-cytoskeleton interactions, allowing nuclear movement driven by dorsal actin cables connected to the nuclear envelope (Fig. 7).

The knowledge on ER shaping proteins role on ER morphology has been growing over the past decade. The role of Kinectin-1 and p180 on ER distribution is still controversial and it might be cell type dependent, but it is noteworthy to mention that the KD of both proteins was described to increase perinuclear ER^41,42^. On the other hand, the role of Climp-63 as a regulator of ER sheets width and perinuclear accumulation is more consensual, and both the luminal and the cytoplasmic domains seem to regulate it^19,21,43^. Climp-63 KD has also been reported to decrease perinuclear ER, which corroborates our results^21^. Our work suggests that the luminal domain of Climp-63 regulates ER morphology and distribution, and nuclear movement in a microtubule-independent fashion.

Nuclear movement in migrating fibroblasts is driven by dorsal actin cables connected to the dorsal side of the nucleus by nesprin-2G on TAN lines^12,15^. Our results show that Climp-63 is not involved on the regulation of dorsal ER. Actin dorsal cables and its connection to the nucleus through TAN lines, the driving force of nuclear movement in migrating fibroblasts, are also not dependent on Climp-63 and ER morphology. Our data suggests that actin-cytoskeleton connections mediated by TAN lines in the dorsal side of the nucleus are independent of ER morphology and any potential interactions between actin and ER. Instead, our results suggest that the ER role on nuclear movement involves the shielding of the immobile ventral stress fibers, thus preventing the connection between the nucleus and ventral stress fibers. We propose a mechanism to inhibit nucleo-cytoskeleton connections involving the ER shielding of actin cytoskeleton. This mechanism generates asymmetric nucleo-cytoskeleton connections required for nuclear movement in the absence of asymmetric distribution of the LINC complex^44^. We hypothesize that this mechanism can also be involved in switching between anchoring and moving nuclei in cells^1,8^, and in mechanotransduction^45^.

The ER contacts multiple organelles and plasma membrane, and these contacts are involved in lipid distribution, calcium regulation and organelle dynamics^46–48^. Here we propose that the ER can also behave as a shield to prevent interactions between actin and the nucleus. We hypothesize the involvement of this ER function in other interactions between cytoskeleton and organelles, and also in ER contacts with organelles.

## Methods

### Cell culture, siRNA transfection, cDNA infection and cDNA microinjection

NIH3T3 fibroblasts (AATC) were cultured in growth medium (DMEM without sodium pyruvate; 41965-039, Life Technologies), supplemented with 10% bovine calf serum (Corning, 35-053-CM), 10 mM HEPES (15630056, Life Technologies), and penicillin/streptomycin at 500 units/ml (15140-122, Life Technologies). U2OS cells (AATC) were cultured in growth medium (DMEM with sodium pyruvate; 41966-029, Life Technologies), supplemented with 10% fetal bovine serum (CVFSVF00-01, Eurobio) and penicillin/streptomycin at 500 units/ml. For both cell types the serum-free medium has the same composition as the growth medium, except for the bovine calf serum or fetal bovine serum. Small interference RNAs (siRNAs) were transfected as previously described^10^ using Lipofectamine RNAiMAX (13778-150, Invitrogen). Nesprin-2G siRNA is custom made from Genecust Europe as previously described^10^. The Scramble (4390843), Climp-63 #1 (s103547), Climp-63 #2 (s103548), p180 (s96290), Kinectin-1 (s69027), Myosin-1C (s70302), and Reticulon-4 (86843) siRNA are commercial Silencer Select siRNAs from ThermoFisher Scientific. The sequences of the siRNAs can be found in Supplementary Table 1. Microinjections of cDNA were performed as described in^15,49^ using a Xenoworks microinjection system (Sutter Instruments). NIH3T3 fibroblasts stable cell lines were created as described in^49^, one only expressing Lifeact-mCherry and a second one coexpressing Lifeact-mCherry and GFP-KDEL. Lentivirus were produced in HEK293T cells (pLALI backbone).

### Wound healing assay and siRNA transfection

Wound healing assay and siRNA transfection were performed as previously described^50^. NIH3T3 fibroblasts were cultured in a 10 cm dish up to 80% confluence. To start the wound healing assay cells were trypsinized and resuspended in 10 ml of growth medium and plated in a 6-well dish. Each well should contain an acid washed coverslip on the bottom and was initially filled with 1750 µl of growth medium (631-0851, VWR). Then, 250 µl of the resuspended cells were added and 500 µl of the siRNA mix, therefore the final volume is 2500 µl per well. The siRNA mix contained the siRNA at 20 nM and 5 µl of lipofectamine per well. Cells were left to grow for 24 h, and after that period the medium was changed to growth medium. After replacing the medium, cells were left to grow for another 24 h. After that period cells were usually between 60 and 75% confluence and were serum-starved for another 48 h with serum-free medium. To induce nuclear movement, 96 h after siRNA transfection, cells were wounded with a 20 µl pipette tip and stimulated with 10 µM LPA (L7260-1MG, Sigma-Aldrich). The LPA stimulation took 2 h in all experiments, except for TAN lines analysis where it took only 50 min (min). After stimulation cells were either fixed for immunofluorescence or used for live imaging. For experiments that involve microinjection, cells were wounded and microinjected with cDNA for 2 h and only after that expression time cells were stimulated with LPA.

### Western Blot for siRNA validation

NIH3T3 fibroblasts seeded on 10 cm dishes were placed on ice and washed twice with PBS at 4 °C. Then, cells were lysed in 300 μL of RIPA buffer composed by 1% Triton X-100, 100 nM NaCl, 10 mM Tris, 1 mM EDTA and Protease Inhibitor Cocktail 1x (1:25, #10085973 Fischer Scientific). Using a cell scrapper, cells were detached from the plate and the lysate was transferred to an ice cold 1.5 mL tube. The cell lysates were then centrifuged at 13 G, for 10 min, at 4 °C and the supernatants collected into a new ice cold 1.5 mL tube. Protein concentration was measured with BCA protein assay kit (Pierce) in a Infinite M200 (Tecan) microplate reader. Equal amount of total protein were loaded in Mini-PROTEAN® TGX 4-15% Precast protein gels (BioRad) and run for 60 min, at 80 Volts. Proteins from the gel were transferred to nitrocellulose membranes using a dry transfer system iBlot Dry Blotting System (Invitrogen). Membranes were probed using primary and secondary antibodies incubated in PBS, 0.1% Tween-20, and 5% milk. The primary antibodies used were rabbit anti-Climp-63 (HPA041143, 1:500, Sigma-Aldrich), rabbit anti-Vinculin (V9131, 1:500, Sigma-Aldrich), rabbit Myosin-1C (HPA001768, 1:1000, Sigma-Aldrich), rabbit Reticulon-4 (HPA023977, 1:1000, Sigma-Aldrich) and mouse Tubulin (T6557, 1:1000, Sigma-Aldrich). Secondary antibodies used were anti-rabbit HRP (Thermo Scientific #31460, 1:5000) and anti-mouse HRP (Thermo Scientific #32430, 1:5000).

### Immunofluorescence

Cells were fixed in 4% paraformaldehyde in PBS for 10 min at room temperature and permeabilized with 0.5% Triton X-100 for 5 min in an orbital shaker. Primary and secondary antibodies were diluted in PBS containing 10% goat serum (G9023-10ML, Sigma-Aldrich). Cells were incubated with primary antibodies overnight at 4 °C in a humidity chamber and then washed three times for 10 min with PBS. Cells were incubated with secondary antibodies for 1 h at room temperature, washed three times for 10 min with PBS, and mounted with Fluoromount-G (Invitrogen). The primary antibodies used for immunofluorescence were: rabbit anti-β-Catenin (712700, 1:200, Invitrogen), mouse anti-Pericentrin (611814, 1:200, BD-Biosciences), rabbit anti-Nesprin-2G (1:200, gift from Gregg Gundersen), rabbit anti-Kinectin-1 (HPA003178, 1:200, Sigma-Aldrich), rabbit anti-p180 (HPA011924, 1:200, Sigma-Aldrich), rat anti-tyrosinated α-tubulin (YL1/2) (1:50, European Collection of Animal Cell Cultures, Salisbury, UK), chicken anti-GFP (GFP-1020, 1:1000, Aves Labs). The anti-rat, anti-mouse, anti-rabbit and anti-chicken secondary antibodies used were Alexa Fluor 488, Alexa Fluor 555 and Alexa Fluor 647 (1:800, Life Technologies). Phalloidin conjugated with Alexa Fluor 488 and Alexa Fluor 555 (A12379 and A34055, respectively, Life Technologies) were used to stain filamentous actin (1:200 dilution). DAPI was used to stain the nucleus (1:10000, Sigma-Aldrich). All of the antibodies and probes that were used are listed in Supplementary Table 2.

### Plasmids

For rescue experiments, mouse Climp-63 cDNA was synthetized (Life Technologies) on a pcDNA3.1 + N-eGFP 6.1Kb expression vector. To test the different Climp-63 regions involved in nuclear positioning five different plasmids were synthetized: Climp-63 full length (Climp-63), transmembrane domain plus cytoplasmic domain (Cyto), transmembrane domain plus luminal domain (Lum), a phospho-mimetic Climp-63 whose microtubule interaction is prevented by mutation of four cytosolic serine residues to glutamic acid (−MT), and a phospho-deficient Climp-63 where the interaction is permanent by mutation of three serines to alanines that cannot be phosphorylated (+MT). Supplementary Table 3 includes the sequences of the synthetized cDNA. All sequences have silent mutations to confer resistance against Climp-63 siRNAs used. All plasmids also included a GFP tag on the N-terminal. The Lifeact-mCherry cDNA used to generate the stable cell line was a gift from Olivier Pertz. The GFP-KDEL used as control was generated in a previous publication^51^. The GFP-mini-N2G, mCherry-mini-N2G and GFP-mN2GΔL were a gift from Gregg Gundersen Lab^12^. The GFP-INF2-CAAX and GFP-INF2-nonCAAX were gifts from Henry Higgs Lab^34^. The HaloTag-Sec61 was a gift from Andrew Moore^52^. Supplementary Table 4 includes all the plasmids used for microinjection.

### FIB-SEM and TEM sample preparation and image acquisition

Cells for FIB-SEM (U2OS) and TEM (NIH3T3) were fixed for 1 h at 4 °C in 0.1 M sodium cacodylate buffer, pH 7.3, containing 2.5% (v/v) glutaraldehyde and 0.1% (v/v) formaldehyde. A post-fixation treatment of 1 h (on ice) in 1% (aq.) osmium tetroxide and 30 min contrast in block in 1% (aq.) uranyl acetate was also performed. Dehydration was made using ethanol gradient (50-70-95-100%). Samples were flat embedded in Durcupan resin and hardened at 60 °C for 72 h.

For TEM imaging, two blocks were glued together to allow sagittal slicing keeping sample integrity. The blocks were sectioned using an ultramicrotome Reichert Supernova (Leica microsystems^©^), Ultra-thin sections (70 nm), were obtained and collected in formvar coated copper slot grids (AGAR scientific^©^), and counter-stained with uranyl acetate and lead citrate (Reynold recipe). It was used a Hitachi H-7650 transmission electron microscope at 100 kV acceleration to image acquisition. For FIB-SEM imaging, images were acquired using a Crossbeam 540 (Carl Zeiss).

### Light microscopy

Light microscopy imaging was performed using a wide variety of imaging techniques and microscopes. For immunofluorescence for siRNA validation, as well as for quantification of nuclear positioning, ventral and dorsal actin, and TAN lines, cells were imaged using an inverted Zeiss Cell Observer widefield microscope controlled by ZEN Blue Edition (Zeiss). For siRNA validation and nuclear positioning, we used a EC Plan-Neofluar ×40/0.75 M27 oil objective, whereas for TAN lines quantification a ×63/1.4 Plan-Apochromat DIC M27 oil objective was used. Zeiss Cell observed was equipped with a sCMOS camera (ORCA-flash4.0 V2, 10 ms/frame streaming acquisition, Hamamatsu), a LED light source Colibri2 (Zeiss).

A Zeiss Cell Observer spinning disk confocal inverted microscope was used for time-lapse imaging analysis of nuclear movement tracking, actin retrograde flow and perinuclear ER accumulation during nuclear positioning. This microscope is equipped with 37 °C chamber and 5% CO_2_ suitable for live-cell microscopy, a EM-CCD camera (Evolve 512, Photometrics), a spinning disc confocal scanner (CSU-x1, Yokogawa) and a LED light source Colibri2 (Zeiss), a ×63 Plan-Apochromat oil objective (NA = 1.4) with a solid state laser 405 nm (maximum power 50 mW) and a 561 nm laser (maximum power 75 mW). Moreover, this microscope has a BP 450/50 425–475 nm emission filter and a BP 600/50 575–625 nm emission filter.

Structural illumination microscopy (SIM) was used for analysis and quantification of ventral actin stress fibers wrapping by ER during nuclear positioning live imaging, as well as to analyze and quantify the area of nuclear ventral ER and colocalization, ventral ER and ventral actin. Ti2-E microscope equipped with an ORCA-Flash 4.0 sCMOS camera (Hamamatsu Photonics), N-SIM E illuminator unit, double layer configuration with Perfect Focus Unit, Piezo Stage, Ti2-FTQ N-SIM Motorized quad band filter turret, Ti2-P-FWB-E Motorized BA filter wheel and lasers unit LU-N3-SIM laser unit 488 nm and 561 nm. The microscope was controlled with NIS-Elements (Nikon) software. An CFI SR HP Apochromat TIRF NA 1.49 ×100C oil objective was used.

Confocal point-scanning microscopy was used to analyze ER morphology, as well as analyze and quantify the area of nuclear ventral ER and colocalization, ventral ER and ventral actin. Two microscope were used, the point-scanning Zeiss LSM 710 (ER morphology) and the LSM 880 (ER morphology, analysis of ventral ER, and quantitative analysis of ventral area of ER and actin colocalization, ER and actin). The Zeiss LSM 710 is equipped with a 63/1.4 Plan-Apochromat DIC M27 oil objective, 37 °C chamber and 5% CO2 for live imaging, diode laser 405-30 (405 nm), argon laser (458, 488 and 514 nm), DPSS 561-10 laser (561 nm) and HeNe633 laser (633 nm) and it is controlled with Zen Black software. The Zeiss LSM 880 is a point-scanning confocal inverted microscope equipped with 37 °C chamber, 5% CO2 and Definite Focus for live-cell microscopy. This microscope is controlled with ZEN Black (Zeiss) and was equipped with a Airyscan detector with 32 channel area (resolution of 140 nm laterally and 400 nm axially, at 488 nm) and a GaAsP detector (photomultiplier 45% QE) and an 488 nm argon laser unit (maximum power of 25 mW). For live imaging a coverslip holder was used (CM-S22-1 and CM-B25-1, Live Cell Instrument Co). We used a 63x Plan-Apochromat Oil NA 1.4 objective with a working distance of 0.19 mm and Z stacks of 220 nm were acquired.

### FIB-SEM data analysis

The images were pre-processed using ImageJ to select regions of interests. Each row was sized around 300 × 300 pixels. A 3D median filter was applied to remove salt and pepper noise before segmentation. To create a 3D model of the intricate structure of the ER we first segmented the structure using the carving algorithm in Ilastik. The segmentation was iteratively redefined and the final mesh was exported and rendered using Blender. The original images were superimposed to the rendered structure using Neuromorph. Each slice was 8 nm thick. The representative images are different slices of the sample, with a 560 nm spatial interval in Z.

### Endoplasmic reticulum volume

FIB-SEM data were quantified using stereology as described in ref. ^53^. To estimate the volume, 18 slices from the complete stack (corresponding to a total volume of 0.60 µm3) were used (1 image every 10 in a 180 slices stack). A grid of test points probes with a random offset was applied to the images using ImageJ, and the number of points landing on the ER were manually counted, and the volume calculated using Cavalieri’s estimator.

### Nuclear positioning analysis

Nuclear positioning was quantified using an automated software developed by Gregg Gundersen’s Lab, Cell Plot (http://www.columbia.edu/~wc2383/software.html). The axis of polarity is coincident with the wound direction. The coordinates of the nuclear and cell centroids are automatically given by the software. The software calculates nuclear positioning as the distance between the nuclear centroid and cell centroid. This distance is represented in box plots with whiskers corresponding to 10 to 90 percentile of the percentage of the cell radius.

### Nuclear roundness, circularity and perimeter analysis

Images of labeled nuclei (DAPI) were segmented manually with image J and corresponding 2D regions were extracted to measure roundness, circularity and perimeter.

Roundness is a measure to capture shape information. Nuclear roundness was computed using the formula: (4 ∗area)/(π*major axis²), where the major axis was computed by fitting an ellipse to the nucleus shape^54^. Nuclear circularity captures perimeter smoothness, and not overall structural shape. It was computed using the formula: 4 *π*(area/perimeter2)^55^. Perimeter was calculated using the length of the outside boundary of the selection (nucleus).

### Nuclear tracking

Nuclear tracking analysis was based on time lapse microscopy. Cells were imaged every 5 min during 120 min of LPA stimulation using a spinning disc. Stable cells expressing GFP-KDEL were used to determine nuclear position in each frame. For nuclear tracking quantification, the Manual Tracking plugin of Image J was used. For this analysis, the coordinates of nucleus centroid were measured in every time point. The nuclear tracking graphs, the persistence, the yy persistence were calculated using Chemotaxis and Migration Tool (Ibidi). Persistence corresponds to directness and YY persistence corresponds to parallel forward migration index in Chemotaxis and Migration Tool.

### Analysis of total ER amount

Individual images of live wound-edge fibroblasts cell expressing GFP-KDEL were imaged between 1h15 and 1h45min after LPA stimulation, in a point scanning Zeiss LSM 880 confocal microscope. A ROI including the total area of the cell was drawn and the mean intensity of GFP-KDEL was quantified.

### Analysis of ER morphology and perinuclear ER accumulation

To analyze ER morphology and perinuclear ER compaction, individual images of live cell expressing GFP-KDEL were imaged between 1h15 and 1h45min after LPA stimulation, in a point scanning Zeiss LSM 880 confocal microscope. For cells not treated with LPA, the imaging was performed 1h45 after wound scratching. Representative images represent a single Z. For both experiments imaging was performed at 37 °C and 5% CO2.

### Analysis of perinuclear ER accumulation during nuclear movement

To analyze perinuclear ER accumulation the KDEL-GFP fluorescence intensity was measured on the ventral, perinuclear and dorsal region of the cell. For both ventral and dorsal regions we selected a Z plane were we could detect the ventral ER and selected a ROI that only included the nuclear region and the fluorescence intensity was measured. To normalize the intensity each fluorescence intensity was normalized to the total intensity of the cell in each timepoint. The same procedure was used to measure ventral and dorsal ER. To measure perinuclear ER, a linescan was traced around the nucleus and each linescan had a thickness of 3 µm. The GFP mean fluorescence intensity was normalized according to the total GFP intensity of the cell in each timepoint.

### Dorsal and ventral actin quantification

To analyze ventral and dorsal filamentous actin, we acquired images of wound edge cells stimulated with LPA for 2 h, fixed and stained with phalloidin-Alexa Fluor 647, focusing on the dorsal and ventral side of cells. We then scored the number of ventral stress fibers and dorsal actin cables per nuclei.

### TAN lines quantification

For TAN lines quantification, WT wound edge starved cells were microinjected with GFP-mini-N2G, and after 2 h of expression, cells were stimulated with LPA for 50 min. Cells were fixed and immunolabelled for GFP and stained for filamentous actin with phalloin-Alexa 647. We quantified the number of TAN lines per nuclei (number of colocalizing lines of Nesprin-2G and dorsal actin cables) and the percentage of nuclei with TAN lines (percentage of nuclei with, at least, one TAN line).

### Actin retrograde flow direction and speed quantification

To analyze actin retrograde flow we performed time lapse microscopy of wound edge cells stably transfected with Lifeact-mCherry, and stimulated with LPA. Images were acquired every 5 m during 2 h. We generated kymographs using rectangular regions perpendicular to the wound edge, from the leading edge to the back of the nucleus (2.3 µm wide).

To calculate actin retrograde flow speed we used trigonometry. Firstly, to calculate the spatial displacement we started by tracing a line on the kymograph from the first timepoint to the last one where we could visualize it continuously. However, the real spatial displacement of the actin cable corresponds to the distance in Y from the first timepoint coordinate to the last timepoint (opposite collet). To calculate the opposite collet we multiplied the hypotenuse (the distance between the first and last timepoint) by the sin of the angle between hypotenuse and the leading edge. The temporal duration, in min, for actin cable displacement corresponds to the number of timepoints where we visualize it multiplied by 5. Moreover, we also quantified the percentage of cells where the flow moved frontward, backwards or did not move at all. Actin cables that moved towards the leading edge were considered to move frontward, actin cables that moved towards the cell rear were considered to move backwards and actin filaments that did not move were considered as stopped.

### Actin shielding by ER during nuclear positioning analysis

To analyze how Climp-63 affected the ER wrapping of actin stress fibers during nuclear positioning, cells coexpressing Lifeact-mCherry and GFP-KDEL were stimulated with LPA and imaged with time lapse microscopy. Cells were stimulated at timepoint zero, and the first frame was acquired at timepoint 60 min. A frame was acquired every 15 min for 60 min. A Z stack of 1 µm with a Z step of 120 nm was acquired each timepoint. Additionally, to analyze the ventral area of ER and actin colocalization, ER and actin under the nucleus cells were imaged after 1 h of LPA stimulation and Z stacks were acquired in single snapshots (LSM 880 and SIM). SIM microscopy experiments were performed using the ×100 objective of Nikon N-SIM E at 37 °C and cells were treated with HEPES at 20 mM to stabilize the pH during acquisition. Image analysis was performed using ImageJ.

The percentage of cells with actin shielding by ER was manually quantified from Z stacks of images of ventral actin and ER. Cells were scored positive when at least an event of ER forming a hook or wrapping around actin stress fibers was observed in at least two consecutive timepoints.

Area of ventral ER and actin colocalization was measured in cells expressing GFP-KDEL or HaloTag-Sec61 labelled with Janelia Fluor^®^ 646 HaloTag^®^ Ligand (Promega, #GA1120) (to label the ER), and LifeAct-mCherry (to label actin filaments). A mask corresponding to the nucleus was used to select ventral ER and actin. ER images were threshold and a binary mask was created. Actin filaments binary masks were manually generated. Overlapping area between ER and actin filaments was calculated with Image Calculator in Image J. The colocalization area was normalized for the area of ventral actin per nucleus. To quantify the percentage of the actin cable covered by ER the ratio between the area of ER and the area of the actin cable was calculated.

### Climp-63 localization analysis

To analyze the localization of Climp-63, Climp-63 siRNA cells were microinjected with HaloTag-Sec61 and Climp-63-GFP. To quantify the area of Climp-63 per total ER area, the ratio between Sec61 area and Climp-63 area was calculated. Threshold method was used to generate the staining masks. The quantification was done both in the ventral region, and the dorsal regions of the cell.

### Nuclear positioning rescue analysis

Rescue experiments were performed to rescue nuclear positioning by microinjecting different constructs into wounded leading edge scramble and Climp-63 depleted cells. Cells were microinjected after 48 h of starvation and cDNA was expressed for 2 h before LPA stimulation. After 2 h of LPA stimulation cells were fixed and stained for nuclear positioning quantification. To test the relevance of the different Climp-63 domains for nuclear positioning, Climp-63 depleted cells were microinjected with KDEL (control), Climp-63 full length, Climp-63 cytoplasmic domain (Cyto), Climp-63 luminal domain (Lum), Climp-63 (−) MT and Climp-63 (+) MT. To test if the effect of Climp-63 depletion was due the loss of ER-actin interacting points, Climp-63 depleted cells were microinjected with GFP-KDEL (control), GFP-mini-N2G lacking the KASH domain (m-N2GΔL), GFP-INF2-CAAX and GFP-INF2-nonCAAX. To guarantee that the siRNA knockdown was efficient in every microinjected coverslip where nuclear positioning was quantified, the nuclear positioning in non-microinjected cells was measured and coverslips where siRNA transfection was inefficient were excluded.

### Data analysis and figure generation

ImageJ FIJI software (available at https://fiji.sc/). Fiji software was used as an imaging processing software and to quantify perinuclear ER accumulation; TAN lines; actin retrograde flow direction; ventral and dorsal actin; area of ventral nuclear ER and actin colocalization, ER and actin; nuclear tracking during nuclear movement; ER luminal width, ER area, nuclear envelope luminal width; Nesprin-2G mean fluorescence. Adobe Illustrator was used to assemble figures’ panels.

### Statistical analysis

Graphpad Prism 8 software was used to analyze and represent the data. Unpaired comparisons were made with Student’s *t*-test, assuming equal variance for both populations and a Gaussian distribution (Two-tailed unpaired t-test). The distribution of data points is expressed as mean ± SEM from three or more independent experiments and the line indicates the mean value. No outliers were removed. Representative images are from at least 3 experiments, except for FIB-SEM and EM imaging where one cell was acquired per treatment in a total of one experiment. *P*‐values are presented as follows: ****p < 0.0001, ***p < 0.001, **p < 0.01, *p < 0.05.

### Reporting summary

Further information on research design is available in the Nature Research Reporting Summary linked to this article.

## Supplementary information

Supplementary Information

Description of Additional Supplementary Files

Supplementary Movie 1

Supplementary Movie 2

Supplementary Movie 3

Supplementary Movie 4

Reporting Summary

## Data Availability

The data that support the findings of this study are available from the corresponding author on reasonable request. All FIB-SEM data are deposited to EMPIAR under accession codes EMPIAR-11002 and EMPIAR-11003. Source data are provided with this paper.

## Source data

Source Data
