# Supplementary material for: Shielding of actin by the endoplasmic reticulum impacts nuclear positioning
Source: Nat Commun. 2022 May 19;13:2763. doi: 10.1038/s41467-022-30388-3 (PMC9120458; doi:10.1038/s41467-022-30388-3)
Supplement: Supplementary file 1 — Supplementary Information [file 41467_2022_30388_MOESM1_ESM.pdf]

Supplementary Information:

Supplementary Figures 1-6

Supplementary Table 1-4

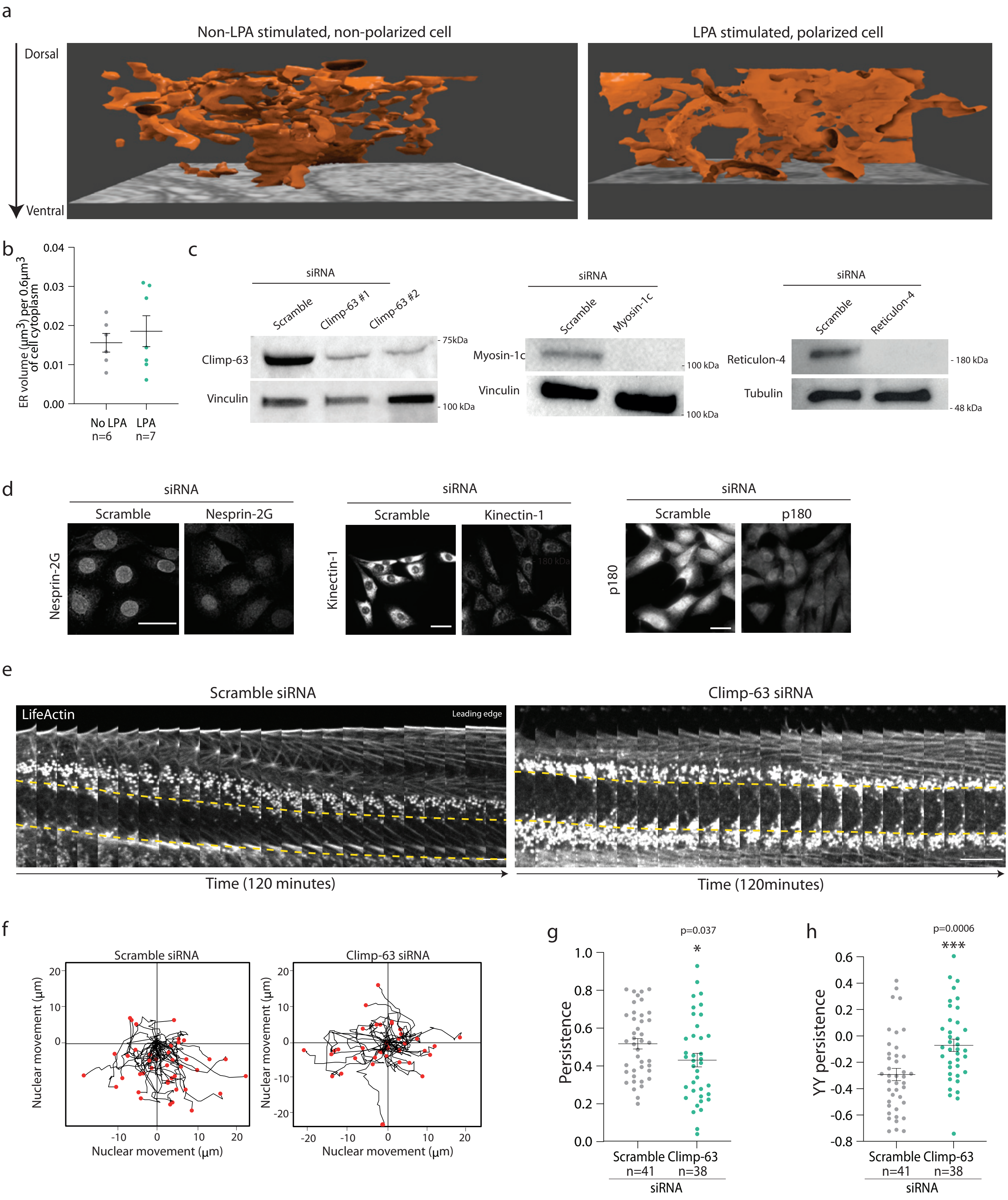
a
Non-LPA stimulated, non-polarized cell
LPA stimulated, polarized cell
Dorsal
Ventral
b
ER volume (µm3) per 0.6µm3 of cell cytoplasm
0.04
0.03
0.02
0.01
0.00
No LPA n=6
LPA n=7
c
siRNA
Scramble
Climp-63 #1
Climp-63 #2
Climp-63
Vinculin
75kDa
100 kDa
Myosin-1c
Vinculin
100 kDa
Reticulon-4
Tubulin
180 kDa
48 kDa
d
siRNA
Scramble
Nesprin-2G
Kinectin-1
p180
e
Scramble siRNA
Climp-63 siRNA
LifeActin
Leading edge
Time (120 minutes)
Time (120minutes)
f
Nuclear movement (µm)
g
Persistence
p=0.037
*
Scramble n=41
Climp-63 n=38
siRNA
h
YY persistence
p=0.0006
***

Supplementary Figure 1.

a. Representative three-dimensional reconstruction of the perinuclear ER of a non-polarized (left) and a polarized cell (right). The dimension of the represented region is 2.4 µm x 2.4 µm x 2.4 µm.

b. Average perinuclear endoplasmic reticulum volume per 0.60 μm^3 of cell cytoplasm, as in cells treated with scramble (grey) or Climp-63 (green) siRNA represented in (a). Data is represented as mean +/- SEM.

c. Representative Western Blot membranes of Climp-63 (n=4) , Myosin-1C (n=3) and Reticulon-4 (n=3) siRNA treated cells and their respective scramble siRNA treated cells.

d. Representative widefield images for siRNA validation of cells treated with Nesprin2-G, Kinectin-1, p180 and their respective control. Cells were stained for each respective protein (Nesprin-2G, Kinectin-1 and p180). Representative 3 independent experiments. Scale bar: 40 µm.

e. Representative kymograph from a spinning disk time-lapse movie during 120 minutes of wound-edge NIH3T3 fibroblasts depicting the nuclear movement upon LPA stimulation (dashed line). Each frame represents 10 minutes. Each frame represents a single Z plane. Representative images of 3 independent experiments. Scale bar: 10 µm.

f. Nuclear movement tracking of a time-lapse movie during 120 minutes depicting the nuclear movement of wound-edge NIH3T3 fibroblasts upon LPA stimulation. A frame was acquired every 5 minutes, in cells treated as in (e).

g. Quantification of nuclear movement persistence in cells treated with scramble (grey) or Climp-63 (green) siRNA as in (f).

h. Quantification of nuclear movement YY persistence in cells treated with scramble (grey) or Climp-63 (green) siRNA as in (f).

Error bars, SEM. Significance (Two-tailed unpaired t-test) was calculated between experimental condition and the scramble siRNA. ***$p < 0.001$, *$p < 0.05$.

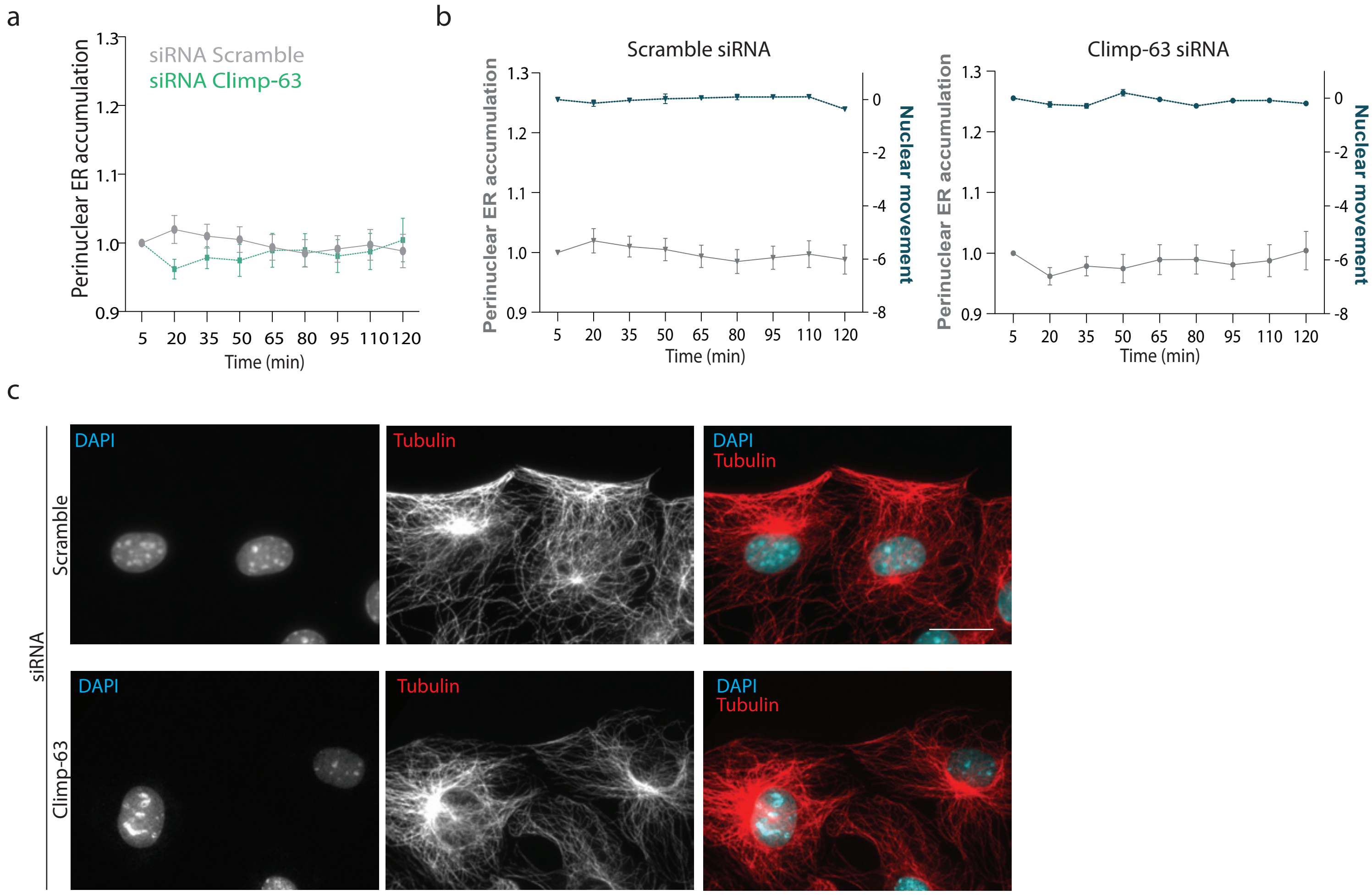
a
siRNA Scramble
siRNA Climp-63
Perinuclear ER accumulation
1.3
1.2
1.1
1.0
0.9
5 20 35 50 65 80 95 110 120
Time (min)
b
Scramble siRNA
Climp-63 siRNA
Perinuclear ER accumulation
Nuclear movement
0
-2
-4
-6
-8
c
siRNA
Scramble
Climp-63
DAPI
Tubulin

Supplementary Figure 2.

a. Quantification of perinuclear ER accumulation in non-LPA stimulated cells treated with scramble (grey, n=30 cells of 3 independent experiments) or Climp-63 (green, n=26 of three independent experiments) siRNA, over time. Data are presented as mean values +/- SEM.

b. Quantification of perinuclear ER accumulation (grey line) and the nuclear movement over time (blue line), in non-stimulated cells in scramble (left, n=52 of 3 independent experiments) or Climp-63 (right, n=50 of 3 independent experiments) siRNA, over time Perinuclear ER accumulation is the same data as in A. Data are presented as mean values +/- SEM.

c. Representative widefield images of wound edge fibroblasts stained with DAPI (blue) and Tubulin (red). Scale bar: 40 μm.

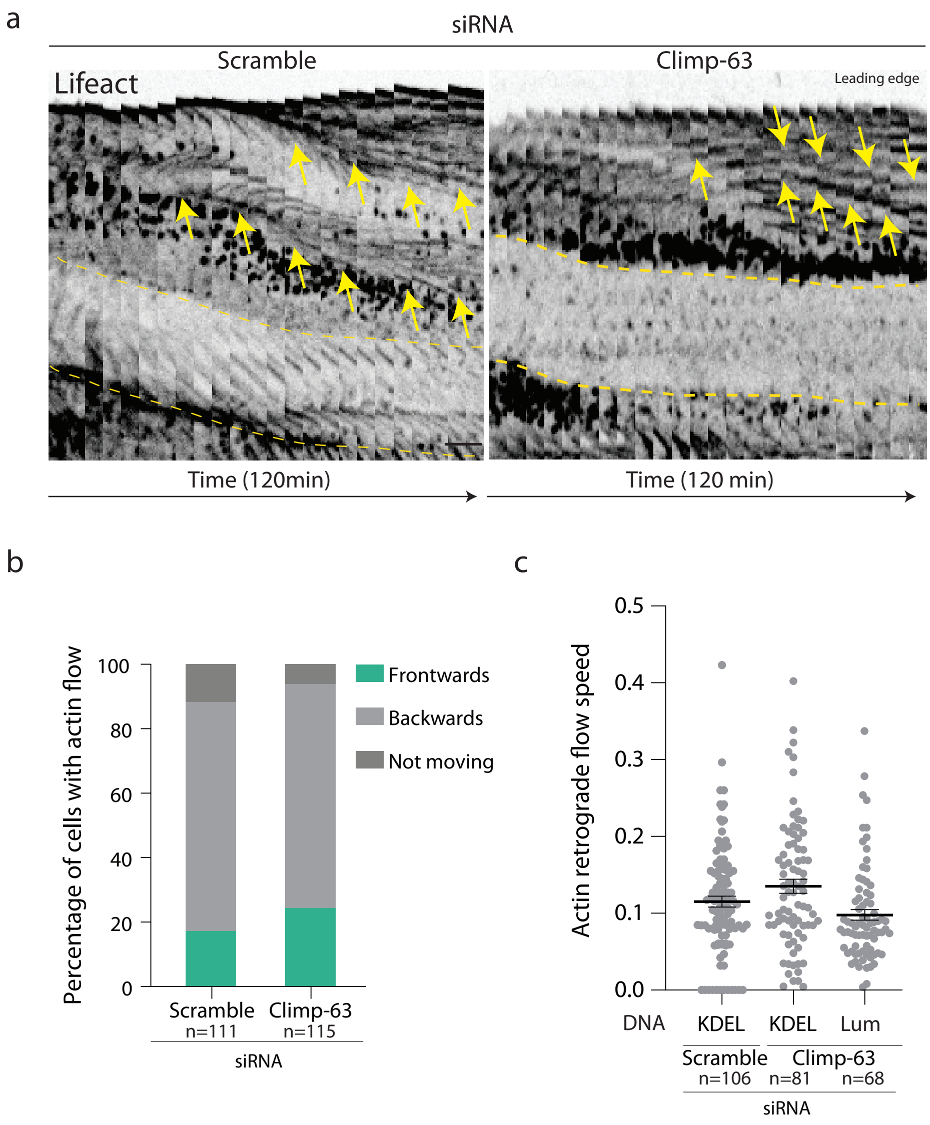
a
siRNA
Scramble
Climp-63
Lifeact
Leading edge
Time (120min)
Time (120 min)
b
Percentage of cells with actin flow
100
80
60
40
20
0
Frontwards
Backwards
Not moving
Scramble
n=111
Climp-63
n=115
siRNA
c
Actin retrograde flow speed
0.5
0.4
0.3
0.2
0.1
0.0
DNA
KDEL
KDEL
Lum
Scramble
Climp-63
n=106
n=81
n=68
siRNA

Supplementary Figure 3.

a. Inverted fluorescence kymograph of actin retrograde flow during LPA stimulation in LifeAct-mCherry expressing wound edge cells. The wound is located at the top. Arrows denote actin cables moving backwards. Dashed yellow line denotes the nucleus. Scale bar: 5 µm.

b. Quantification of percentage of cells with actin cables not moving, moving frontwards or backwards in cells treated as in (a).

c. Quantification of actin retrograde flow speed in scramble siRNA cells overexpressing GFP-KDEL, and Climp-63 siRNA cells overexpressing GFP-KDEL or the luminal domain of Climp-63 (Lum). Data are presented as mean values +/- SEM.

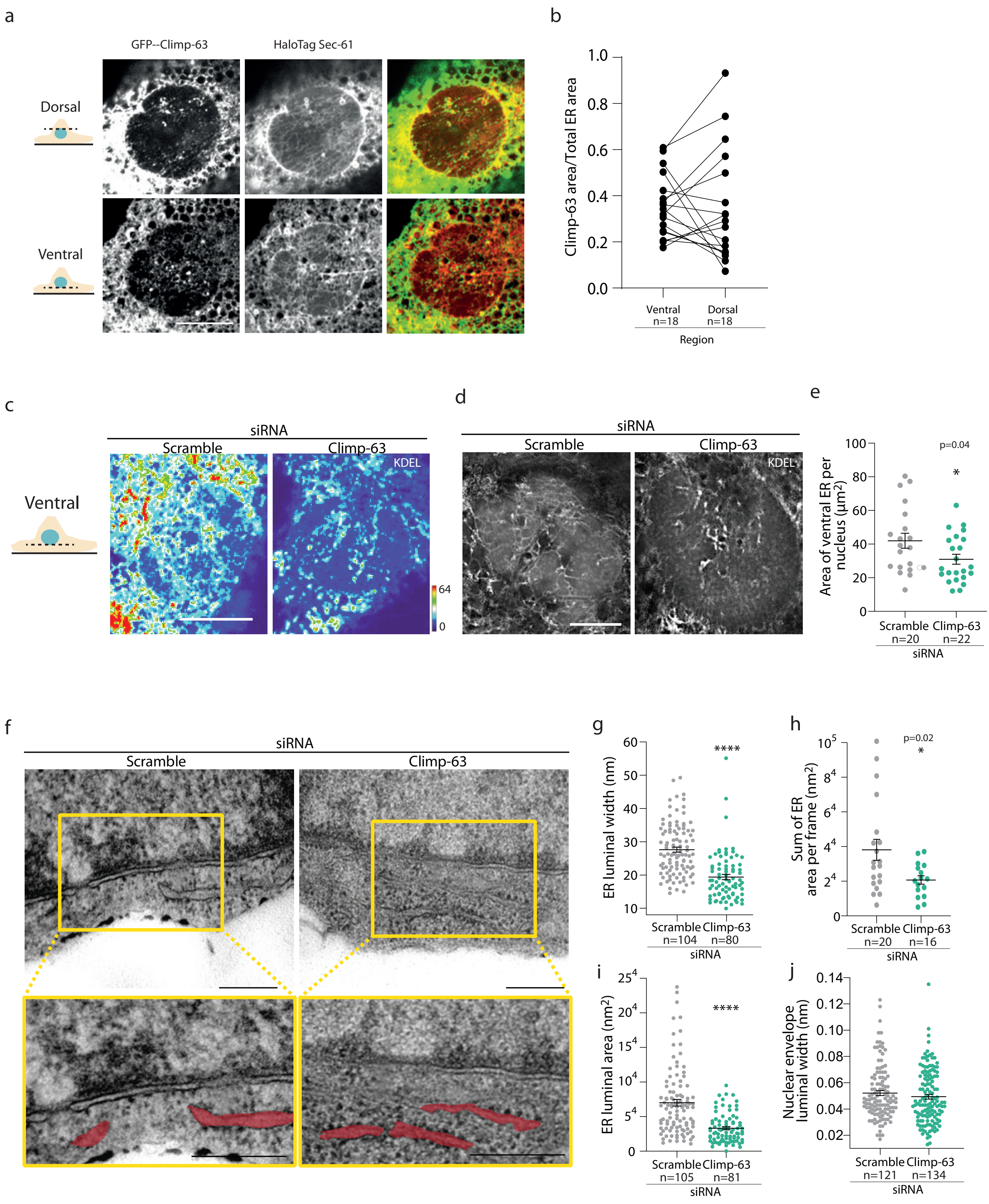
a
GFP--Climp-63
HaloTag Sec-61
Dorsal
Ventral
b
Climp-63 area/Total ER area
1.0
0.8
0.6
0.4
0.2
0.0
Ventral n=18
Dorsal n=18
Region
c
siRNA
Scramble
Climp-63
KDEL
Ventral
64
0
d
siRNA
Scramble
Climp-63
KDEL
e
Area of ventral ER per nucleus (μm2)
100
80
60
40
20
0
p=0.04
*
Scramble n=20
Climp-63 n=22
siRNA
f
siRNA
Scramble
Climp-63
g
ER luminal width (nm)
60
50
40
30
20
10
****
Scramble n=104
Climp-63 n=80
siRNA
h
Sum of ER area per frame (nm2)
10^5
8^4
6^4
4^4
2^4
0
p=0.02
*
Scramble n=20
Climp-63 n=16
siRNA
i
ER luminal area (nm2)
25^4
20^4
15^4
10^4
5^4
0
****
Scramble n=105
Climp-63 n=81
siRNA
j
Nuclear envelope luminal width (nm)
0.14
0.12
0.10
0.08
0.06
0.04
0.02
Scramble n=121
Climp-63 n=134
siRNA

Supplementary Figure 4.

a. Representative confocal images of Climp-63 localization in wound-edge LPA stimulated cells treated Climp-63 siRNA, overexpressing an ER marker (HaloTag Sec-61), and Climp-63 (GFP-Climp-63). Scale bar: 10 µm.

b. Quantification of the ratio of Climp-63 area per total ER area per cell, as represented in A. A matched-pair analysis (ventral and dorsal) is presented for each cell.

c. Representative confocal airyscan images of scramble and Climp-63 siRNA treated cells expressing GFP-KDEL. Scale bar: 10 µm. Images are representative of three independent experiments.

d. Representative structural illumination microscopy (SIM) images of cells expressing GFP-KDEL treated with scramble and Climp-63 siRNA. Ventral region of the cell. Scale bar: 5 µm. Images are representative of three independent experiments.

e. Quantification of the area of ventral ER per nucleus in cells treated with scramble (grey) or Climp-63 (green) siRNA as in (d).

f. Representative transmission electron microscopy images of sagittal sections of scramble and Climp-63 siRNA treated wound-edge cells stimulated with LPA. The regions of interest highlighted (yellow box) are zoomed in on the bottom. ER is highlighted in red. Images are representative of one experiment. Scale bar: 250 nm.

g. Quantification of the ER luminal width, in cells treated with scramble (grey) or Climp-63 (green) siRNA as in (F). Three luminal widths along each ER individual ER unit were measured and averaged, so each point corresponds to the average luminal width per ER unit.

h. Quantification of the sum of ER area per frame lines, in cells treated with scramble (grey) or Climp-63 (green) siRNA as in (f).

i. Quantification of the ER individual units' area per frame, in cells treated with scramble (grey) or Climp-63 (green) siRNA as in (f).

j. Quantification of nuclear envelope luminal width, in cells treated with scramble (grey) or Climp-63 (green) siRNA as in(F). Three nuclear envelope widths along the nuclear envelope were measured and averaged, so each point corresponds to the average nuclear envelope spacing per frame. Scale bar = 250 nm. Bars represent mean +/- SEM. Significance (Two-tailed unpaired t-test) was calculated between experimental condition and the scramble siRNA. **** $p < 0.0001$, *$p < 0.05$.

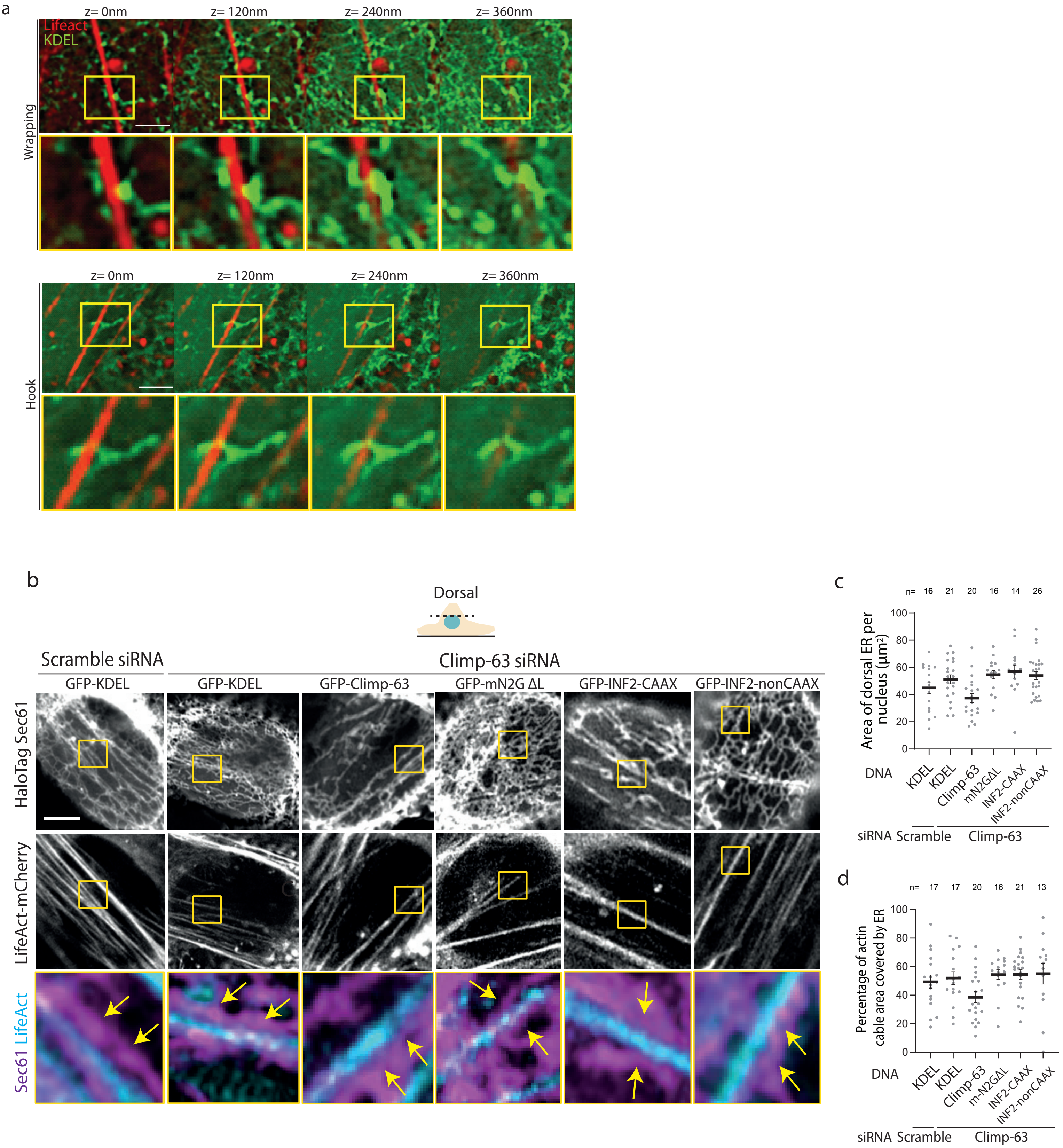

a
z= 0nm
z= 120nm
z= 240nm
z= 360nm
Lifeact
KDEL
Wrapping
Hook
b
Dorsal
Scramble siRNA
Climp-63 siRNA
GFP-KDEL
GFP-KDEL
GFP-Climp-63
GFP-mN2G ΔL
GFP-INF2-CAAX
GFP-INF2-nonCAAX
HaloTag Sec61
LifeAct-mCherry
Sec61 LifeAct
c
n= 16 21 20 16 14 26
Area of dorsal ER per nucleus (μm²)
DNA
KDEL
KDEL
Climp-63
mN2GΔL
INF2-CAAX
INF2-nonCAAX
siRNA Scramble
Climp-63
d
n= 17 17 20 16 21 13
Percentage of actin cable area covered by ER
DNA
KDEL
KDEL
Climp-63
m-N2GΔL
INF2-CAAX
INF2-nonCAAX
siRNA Scramble
Climp-63

Supplementary Figure 5

a. Representative SIM multiple z-plane images of GFP-KDEL (green) and LifeAct-mCherry (red) expressing wound-edge cells depicting the ER wrapping around ventral actin stress fibers (top) or the ER forming a hook around ventral actin stress fibers (bottom). Scale bar = 1 μm.

b. Representative confocal Airyscan images of wound-edge cells stably expressing LifeActin-mCherry, and microinjected with HaloTag Sec-61 together with GFP-KDEL, GFP-Climp-63 or GFP-mN2GΔL, GFP-INF2-CAAX and GFP-INF2-nonCAAX. The focal plane represents the dorsal region of the nucleus. The highlighted ROI (yellow box) represent the insets presented below. Scale bar: 5 μm.

c. Quantification of area of dorsal ER in cells treated as in (b). Error bars, SEM.

d. Quantification of percentage of actin cables's area covered by ER in cells treated as in (b). Error bars, SEM.

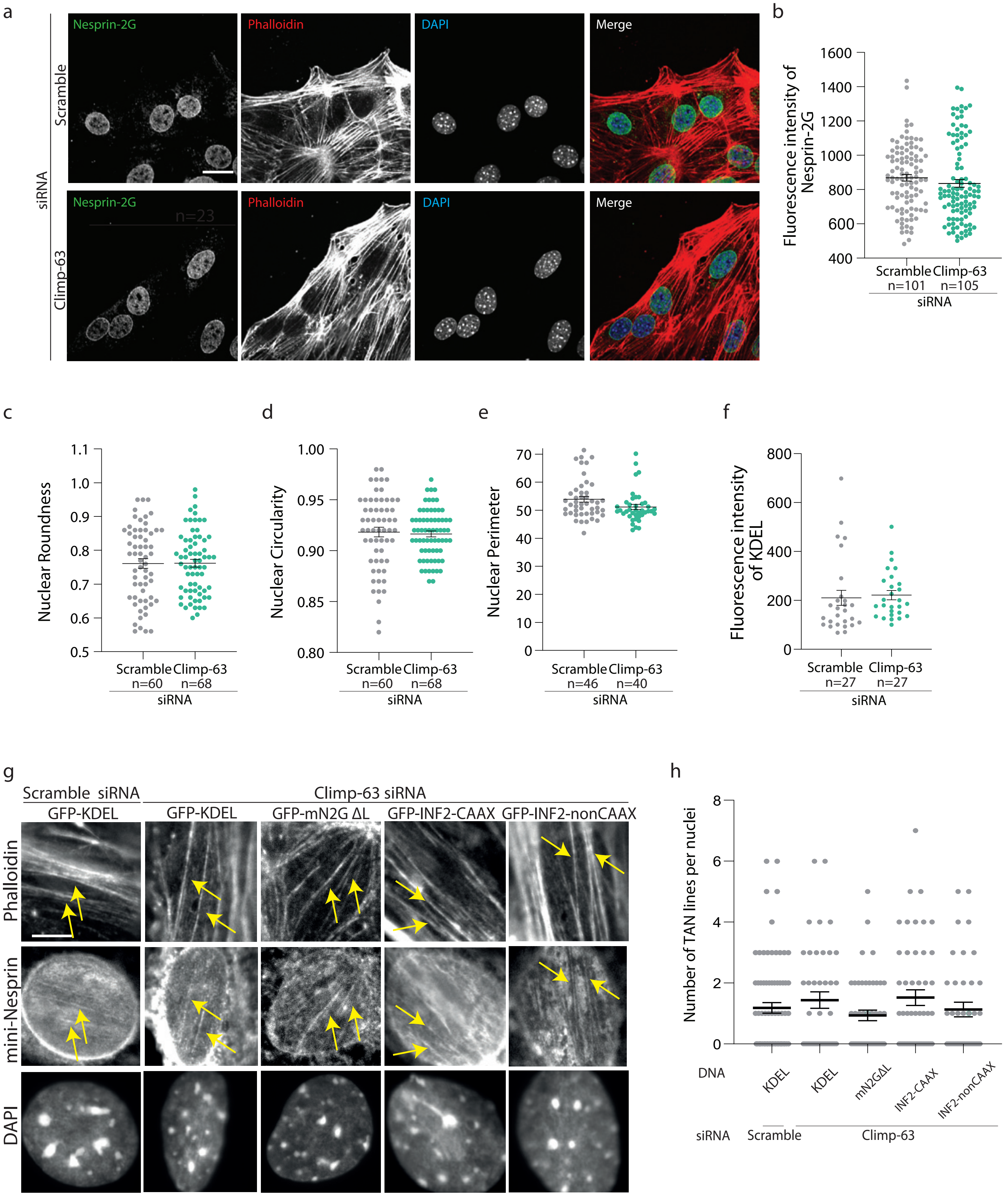
a
siRNA
Scramble
Climp-63
Nesprin-2G
Phalloidin
DAPI
Merge
n=23
b
Fluorescence intensity of Nesprin-2G
1600
1400
1200
1000
800
600
400
Scramble
n=101
Climp-63
n=105
siRNA
c
Nuclear Roundness
1.1
1.0
0.9
0.8
0.7
0.6
0.5
Scramble
n=60
Climp-63
n=68
siRNA
d
Nuclear Circularity
1.00
0.95
0.90
0.85
0.80
Scramble
n=60
Climp-63
n=68
siRNA
e
Nuclear Perimeter
70
60
50
40
30
20
10
0
Scramble
n=46
Climp-63
n=40
siRNA
f
Fluorescence intensity of KDEL
800
600
400
200
0
Scramble
n=27
Climp-63
n=27
siRNA
g
Scramble siRNA
Climp-63 siRNA
GFP-KDEL
GFP-KDEL
GFP-mN2G ΔL
GFP-INF2-CAAX
GFP-INF2-nonCAAX
Phalloidin
mini-Nesprin
DAPI
h
Number of TAN lines per nuclei
8
6
4
2
0
DNA
KDEL
KDEL
mN2GΔL
INF2-CAAX
INF2-nonCAAX
siRNA
Scramble
Climp-63

Supplementary Figure 6

a. Epifluorescence images of wound-edge fibroblast stimulated with LPA. Cells were stained for Nesprin-2G (green), filamentous actin (red) and DAPI (blue). Scale bar = 20 μm. Images are representative of three independent experiments and represent single Z.

b. Quantification of nuclear fluorescence intensity of Nesprin-2G antibody in cells treated with scramble (grey) or Climp-63 (green) siRNA as in (a).

c. Quantification of nuclear roundness in cells treated with scramble (grey) or Climp-63 (green) siRNA as in (a).

d. Quantification of nuclear circularity in cells treated with scramble (grey) or Climp-63 (green) siRNA as in (a).

e. Quantification of nuclear perimeter in cells treated with scramble (grey) or Climp-63 (green) siRNA as in (a).

f. Quantification of overall KDEL-GFP (ER marker) intensity in wound-edge LPA stimulated cells treated with scramble (grey) or Climp-63 (green) siRNA. Data is represented as mean +/- SEM.

g. Representative wide-field images of wound-edge Scramble and Climp-63 siRNA cells overexpressing mCherry-mini-Nesprin2G together with GFP-KDEL, GFP-m-N2GΔL, GFP-INF2-CAAX or GFP-INF2-nonCAAX. Cells were stained for actin (phalloidin), and nucleus (DAPI). Scale bar: 5 μm.

h. Quantification of the number of TAN lines in cells treated as in (g).
Experiments were repeated ≥3. Error bars, SEM.

Supplementary Tables

Supplementary table 1. The siRNAs sequences used in the manuscript.

siRNA Protein	siRNA sequence
Nesprin-2G	5'-CCAUCAUCCUGCACUUUCATT-3'
Climp-63 #1	5'-GCUCAACCGAAUUAGCGAATT-3'
Climp-63 #2	5'-CCGUCAAAAUCGAAACGAATT-3'
Kinectin-1	5'-GUCUAAAAAUGGAAGCGAATT-3'
P180	5'-CAGAUACAGUUGCUAAUCATT-3'
Myosin-1c	5'-CAUGUGAUCGAGACACUAATT-3'
Reticulon-4	5'GUGUUGAUGUGGGUAUUUATT-3'

Supplementary table 2. The antibodies and fluorescent probes used in the manuscript.

Antibody/Probe	Source	Reference	Dilution
Rabbit anti-β-Catenin	Invitrogen	#712700	1:200
Rat anti-Tubulin	Home made	-	1:50
Mouse anti-Pericentrin	BD-Biosciences	#611814	1:200
Rabbit anti-Nesprin-2G	Home made	-	1:50
Chicken anti-GFP	Aves Lab	#GFP-1020	1:1000
Goat anti-Chicken IgY Alexa Fluor® 488 conjugate	ThermoFisher Scientific	#A-11039	1:600
Donkey Anti-Mouse IgG Alexa Fluor® 488 conjugate	ThermoFisher Scientific	#A21202	1:600
Donkey Anti-Rabbit IgG Alexa Fluor® 488 conjugate	ThermoFisher Scientific	#A21206	1:600
Alexa Fluor® 488 and 647 Phalloidin	Life Technologies	#A12379<br>#A22287	1:200
DAPI	Sigma-Aldrich	#32670-5MG-F	1:10000

Supplementary table 3. Sequences used for gene synthesis of Climp-63 plasmids.

Description	Gene synthesis sequence
GFP-Climp63 Full Length siRNA resistant	GAATTCacaagtTTGTACAAAAAAGCAGGCtcaggaatgccctcggccaaacaaaggggctccaagggcggccacggcgccgcgagcccctcggacaagggcgcccacccgtcgggcg<br>gcgcggatgacgtggcgaagaagccgccggcggcgccgcagcagccgcagcctcccgcgccgcacccgccgcagcacccgcagaaccaggcgcaccggggcggccaccgcggccggtcctcagc<br>cgccaccgccaacgcgtcctccgcgtcctgctcgcgcaggcttggccgggtgctcaactttctcttctacctctccctggtggcggcggccgccttctcgggctggtatgtccatcacgtcctggaggagg<br>tccagcaggtccggcgtggccaccaggacttctcccgtcagagggatgagctgggccagggcttgcagggcgtcgagcagaaggtccaatccctgcaagccacatttgggactttcgagtccctcctg<br>agaaactcccagcataaacaggatctcacagagaaagccgtgaaggaaggggagagcGAACTAAATCGTATAAGTGAGgttctacagaagttgcagaatgagattctcaaagatctgtc<br>ggacgggatccacgtggtgaaggatgcccgggagcgggacttcacgtccctggagaacacagtcgaggagcgcctgacggagcttaccaagtctatcaatgacaacatcgccatcttcactgatgtc<br>cagaagaggagccagaaggagatcaatgaggtcaagatgaaggtggcctccctggaagaatccaaggggggatcgcagtcaggacgtgaaaactctgaaggacgccgtgaaggaggtccaggcct<br>ccatgatgtcgagggagagggacattgaggccctgaagagttcccttcagacgatggagtccgacgtctacacagaggtgcgggaactggtgagcctcaagcaggagcaacaggcgttcaagcag<br>gcggccgactcagagcgcctggctctgcaggccctcacggagaagcttctgcggtccgaggagtcctcctctcgtctcccagaggacatccggagactggaggaagagctacagcagctgaaggtc<br>ggggcccatgggtcagaggaaggtgccgtcttcaaagactccaaagccttagaggaactgcagaggcagattgagggcctgggtgccaggctccagtatgtagaggacggggtctactcaatgcag<br>gtggcttctgcacgccacaccgaaagcctggagtcgctcctgtccaagagccaggagtacgagcaacgcctggctatgctgcaggagcacgtgggaaacctgggctcctcatctgacctggccagca<br>cagtcaggagcctgggggagacccagctggcgctgtccagcgacttgaaggagctgaagcagagcctgggtgagctgcccggtaccgtggagtcactgcaggagcaagtgctctcactgctcagcc<br>aggaccaagcccaggccgagggcttgcctcctcaggacttcctggacagactgtcctctctagacaacctgaaatcctcagtgagccaagtggagtcagacttgaaaatgctcaggactgccgtgga<br>cagtttggtggcctattccgtcaaaatcgaaacgaatgagaataacttagagtcagccaagggcttgctggatgacctgcggaacgacctggataggttgtttctgaaagttgagaagatccatgaaa<br>agatctaaacCCAGCTTTCTTGTACAAAGTGGtCTCGAG
GFP-Climp63 Cytoplasmic siRNA resistant	GAATTCacaagtTTGTACAAAAAAGCAGGCtcaggaatgccctcggccaaacaaaggggctccaagggcggccacggcgccgcgagcccctcggacaagggcgcccacccgtcgggcg<br>gcgcggatgacgtggcgaagaagccgccggcggcgccgcagcagccgcagcctcccgcgccgcacccgccgcagcacccgcagaaccaggcgcaccggggcggccaccgcggccggtcctcagcc<br>gccaccgccaacgcgtcctccgcgtcctgctcgcgcaggcttggccgggtgctcaactttctcttctacctctccctggtggcggcggccgccttctcgggctggtatgtccatcactaaacCCAGCTTT<br>CTTGTACAAAGTGGtCTCGAG
GFP-Climp63 Luminal siRNA resistant	GAATTCacaagtTTGTACAAAAAAGCAGGCtcaggaatggtgctcaactttctcttctacctctccctggtggcggcggccgccttctcgggctggtatgtccatcacgtcctggaggaggtc<br>cagcaggtccggcgtggccaccaggacttctcccgtcagagggatgagctgggccagggcttgcagggcgtcgagcagaaggtccaatccctgcaagccacatttgggactttcgagtccctcctga<br>gaaactcccagcataaacaggatctcacagagaaagccgtgaaggaaggggagagcGAACTAAATCGTATAAGTGAGgttctacagaagttgcagaatgagattctcaaagatctgtcg<br>gacgggatccacgtggtgaaggatgcccgggagcgggacttcacgtccctggagaacacagtcgaggagcgcctgacggagcttaccaagtctatcaatgacaacatcgccatcttcactgatgtcc<br>agaagaggagccagaaggagatcaatgaggtcaagatgaaggtggcctccctggaagaatccaaggggggatcgcagtcaggacgtgaaaactctgaaggacgccgtgaaggaggtccaggcctc<br>catgatgtcgagggagagggacattgaggccctgaagagttcccttcagacgatggagtccgacgtctacacagaggtgcgggaactggtgagcctcaagcaggagcaacaggcgttcaagcagg<br>cggccgactcagagcgcctggctctgcaggccctcacggagaagcttctgcggtccgaggagtcctcctctcgtctcccagaggacatccggagactggaggaagagctacagcagctgaaggtcg<br>gggcccatgggtcagaggaaggtgccgtcttcaaagactccaaagccttagaggaactgcagaggcagattgagggcctgggtgccaggctccagtatgtagaggacggggtctactcaatgcagg<br>tggcttctgcacgccacaccgaaagcctggagtcgctcctgtccaagagccaggagtacgagcaacgcctggctatgctgcaggagcacgtgggaaacctgggctcctcatctgacctggccagcac<br>agtcaggagcctgggggagacccagctggcgctgtccagcgacttgaaggagctgaagcagagcctgggtgagctgcccggtaccgtggagtcactgcaggagcaagtgctctcactgctcagcca<br>ggaccaagcccaggccgagggcttgcctcctcaggacttcctggacagactgtcctctctagacaacctgaaatcctcagtgagccaagtggagtcagacttgaaaatgctcaggactgccgtggac<br>agtttggtggcctattccgtcaaaatcgaaacgaatgagaataacttagagtcagccaagggcttgctggatgacctgcggaacgacctggataggttgtttctgaaagttgagaagatccatgaaaa<br>gatctaaacCCAGCTTTCTTGTACAAAGTGGtCTCGAG
GFP-Climp63 (-)MT siRNA resistant	GAATTCacaagtTTGTACAAAAAAGCAGGCtcaggaatgcccgaagccaaacaaaggggctccaagggcggccacggcgccgcggaacccgaagacaagggcgcccacccgtcgggc<br>ggcgcggatgacgtggcgaagaagccgccggcggcgccgcagcagccgcagcctcccgcgccgcacccgccgcagcacccgcagaaccaggcgcaccggggcggccaccgcggccggtcctcag<br>ccgccaccgccaacgcgtcctccgcgtcctgctcgcgcaggcttggccgggtgctcaactttctcttctacctctccctggtggcggcggccgccttcgaaggctggtatgtccatcacgtcctggagga<br>ggtccagcaggtccggcgtggccaccaggacttctcccgtcagagggatgagctgggccagggcttgcagggcgtcgagcagaaggtccaatccctgcaagccacatttgggactttcgagtccctc<br>ctgagaaactcccagcataaacaggatctcacagagaaagccgtgaaggaaggggagagcGAACTAAATCGTATAAGTGAGgttctacagaagttgcagaatgagattctcaaagatct<br>gtcggacgggatccacgtggtgaaggatgcccgggagcgggacttcacgtccctggagaacacagtcgaggagcgcctgacggagcttaccaagtctatcaatgacaacatcgccatcttcactgat<br>gtccagaagaggagccagaaggagatcaatgaggtcaagatgaaggtggcctccctggaagaatccaaggggggatcgcagtcaggacgtgaaaactctgaaggacgccgtgaaggaggtccag<br>gcctccatgatgtcgagggagagggacattgaggccctgaagagttcccttcagacgatggagtccgacgtctacacagaggtgcgggaactggtgagcctcaagcaggagcaacaggcgttcaag<br>caggcggccgactcagagcgcctggctctgcaggccctcacggagaagcttctgcggtccgaggagtcctcctctcgtctcccagaggacatccggagactggaggaagagctacagcagctgaag<br>gtcggggcccatgggtcagaggaaggtgccgtcttcaaagactccaaagccttagaggaactgcagaggcagattgagggcctgggtgccaggctccagtatgtagaggacggggtctactcaatg<br>caggtggcttctgcacgccacaccgaaagcctggagtcgctcctgtccaagagccaggagtacgagcaacgcctggctatgctgcaggagcacgtgggaaacctgggctcctcatctgacctggcca<br>gcacagtcaggagcctgggggagacccagctggcgctgtccagcgacttgaaggagctgaagcagagcctgggtgagctgcccggtaccgtggagtcactgcaggagcaagtgctctcactgctca<br>gccaggaccaagcccaggccgagggcttgcctcctcaggacttcctggacagactgtcctctctagacaacctgaaatcctcagtgagccaagtggagtcagacttgaaaatgctcaggactgccgt<br>ggacagtttggtggcctattccgtcaaaatcgaaacgaatgagaataacttagagtcagccaagggcttgctggatgacctgcggaacgacctggataggttgtttctgaaagttgagaagatccatg<br>aaaagatctaaacCCAGCTTTCTTGTACAAAGTGGtCTCGAG
GFP-Climp63 (+)MT siRNA resistant	GAATTCacaagtTTGTACAAAAAAGCAGGCtcaggaatgcccgctgccaaacaaaggggctccaagggcggccacggcgccgcggctcccgctgacaagggcgcccacccgtcgggcg<br>gcgcggatgacgtggcgaagaagccgccggcggcgccgcagcagccgcagcctcccgcgccgcacccgccgcagcacccgcagaaccaggcgcaccggggcggccaccgcggccggtcctcagc<br>cgccaccgccaacgcgtcctccgcgtcctgctcgcgcaggcttggccgggtgctcaactttctcttctacctctccctggtggcggcggccgccttctcgggctggtatgtccatcacgtcctggaggagg<br>tccagcaggtccggcgtggccaccaggacttctcccgtcagagggatgagctgggccagggcttgcagggcgtcgagcagaaggtccaatccctgcaagccacatttgggactttcgagtccctcctg

	agaaactcccagcataaacaggatctcacagagaaagccgtgaaggaaggggagagcGAACTAAATCGTATAAGTGAGgttctacagaagttgcagaatgagattctcaaagatctgtc<br>ggacgggatccacgtggtgaaggatgcccgggagcgggacttcacgtccctggagaacacagtcgaggagcgcctgacggagcttaccaagtctatcaatgacaacatcgccatcttcactgatgtc<br>cagaagaggagccagaaggagatcaatgaggtcaagatgaaggtggcctccctggaagaatccaagggggatcgcagtcaggacgtgaaaactctgaaggacgccgtgaaggaggtccaggcct<br>ccatgatgtcgagggagagggacattgaggccctgaagagttcccttcagacgatggagtccgacgtctacacagaggtgcgggaactggtgagcctcaagcaggagcaacaggcgttcaagcag<br>gcggccgactcagagcgcctggctctgcaggccctcacggagaagcttctgcggtccgaggagtcctcctctcgtctcccagaggacatccggagactggaggaagagctacagcagctgaaggtc<br>ggggcccatgggtcagaggaaggtgccgtcttcaaagactccaaagccttagaggaactgcagaggcagattgagggcctgggtgccaggctccagtatgtagaggacggggtctactcaatgcag<br>gtggcttctgcacgccacaccgaaagcctggagtcgctcctgtccaagagccaggagtacgagcaacgcctggctatgctgcaggagcacgtgggaaacctgggctcctcatctgacctggccagca<br>cagtcaggagcctgggggagacccagctggcgctgtccagcgacttgaaggagctgaagcagagcctgggtgagctgcccggtaccgtggagtcactgcaggagcaagtgctctcactgctcagcc<br>aggaccaagcccaggccgagggcttgcctcctcaggacttcctggacagactgtcctctctagacaacctgaaatcctcagtgagccaagtggagtcagacttgaaaatgctcaggactgccgtgga<br>cagtttggtggcctattccgtcaaaatcgaaacgaatgagaataacttagagtcagccaagggcttgctggatgacctgcggaacgacctggataggttgtttctgaaagttgagaagatccatgaaa<br>agatctaaacCCAGCTTTCTTGTACAAAGTGGtCTCGAG

Supplementary table 4. The plasmids used for in the manuscript for microinjection.

Description	Plasmid	Origin
KDEL	GFP-KDEL	(23)
Climp-63 full length	GFP-Climp-63	This project
Climp-63 luminal domain with transmembrane domain	GFP-Climp-63-Lum	This project
Climp-63 cytoplasmic domain with transmembrane domain	GFP-Climp-63-Cyto	This project
Climp-63 that does not bind to microtubules	GFP-MT-(-)	This project
Climp-63 that constitutively binds to microtubules	GFP-MT-(+)	This project
Short version of Nesprin2G with actin binding domain and KASH domain	EGFP-mini-N2G	(4)
The same as GFP-mini-N2G lacking the luminal region of KASH domain	EGFP-m-N2GΔL	Gift from Gregg Gundersen, Columbia University
INF2 isoform that binds to ER	GFP-INF2-CAAX	Gift from Henry Higgs (18)
Cytoplasmic isoform of INF2 that does not bind to ER	GFP-INF2-nonCAAX	
Sec-61 with HaloTag	HaloTag Sec-61	Gift from Andrew Moore, Janelia Research Campus
