# Supplementary material for: Shielding of actin by the endoplasmic reticulum impacts nuclear positioning
Source: Nat Commun. 2022 May 19;13:2763. doi: 10.1038/s41467-022-30388-3 (PMC9120458; doi:10.1038/s41467-022-30388-3)
Supplement: Supplementary file 2 — Description of Additional Supplementary Files [file 41467_2022_30388_MOESM2_ESM.pdf]

Description of Additional Supplementary Files

File Name: Supplementary Movie 1
Description: Perinuclear ER accumulation during nuclear positioning. Time-lapse fluorescence movie of LPAstimulated wound edge GFP-KDELexpressing cell treated with scramble siRNA (the same cell presented in Fig. 2B, Top). Wound edge is towards the top. The signal intensity is represented as a thermal heatmap.

File Name: Supplementary Movie 2
Description: Climp-63 depletion blocks perinuclear ER accumulation during nuclear positioning. Time-lapse fluorescence movie of LPAstimulated wound edge GFP-KDELexpressing cell treated with Climp-63 siRNA (the same cell presented in Fig. 2B, Bottom). Wound edge is towards the top. The signal intensity is represented as a thermal heatmap.

File Name: Supplementary Movie 3
Description: Ventral stress fibers wrapped up by ER. Sequential Z-plane SIM movie of ventral area of GFP-KDEL (green) and LifeAct-mCherry (red) expressing wound-edge cells depicting a wrapping event of ER around a ventral stress fiber (same cell is presented in Sup. Fig. 4A, top).

File Name: Supplementary Movie 4
Description: Ventral stress fibers wrapped up by ER, with a hook shape. Sequential Z-plane SIM movie of ventral area of GFP-KDEL (green) and LifeAct-mCherry (red) expressing wound-edge cells depicting ER with a hook shape around a ventral stress fiber (same cell is presented in Sup. Fig. 4A, bottom).
