# Supplementary material for: Shielding of actin by the endoplasmic reticulum impacts nuclear positioning
Source: Nat Commun. 2022 May 19;13:2763. doi: 10.1038/s41467-022-30388-3 (PMC9120458; doi:10.1038/s41467-022-30388-3)
Supplement: Supplementary file 7 — Reporting Summary [file 41467_2022_30388_MOESM7_ESM.pdf]

nature research

Corresponding author(s): Edgar R. Gomes

Last updated by author(s): Apr 9, 2022

Reporting Summary

Nature Research wishes to improve the reproducibility of the work that we publish. This form provides structure for consistency and transparency in reporting. For further information on Nature Research policies, see our Editorial Policies and the Editorial Policy Checklist.

Statistics

For all statistical analyses, confirm that the following items are present in the figure legend, table legend, main text, or Methods section.

n/a	Confirmed	
☐	☒	The exact sample size (*n*) for each experimental group/condition, given as a discrete number and unit of measurement
☐	☒	A statement on whether measurements were taken from distinct samples or whether the same sample was measured repeatedly
☐	☒	The statistical test(s) used AND whether they are one- or two-sided<br>*Only common tests should be described solely by name; describe more complex techniques in the Methods section.*
☒	☐	A description of all covariates tested
☒	☐	A description of any assumptions or corrections, such as tests of normality and adjustment for multiple comparisons
☐	☒	A full description of the statistical parameters including central tendency (e.g. means) or other basic estimates (e.g. regression coefficient) AND variation (e.g. standard deviation) or associated estimates of uncertainty (e.g. confidence intervals)
☐	☒	For null hypothesis testing, the test statistic (e.g. *F*, *t*, *r*) with confidence intervals, effect sizes, degrees of freedom and *P* value noted<br>*Give P values as exact values whenever suitable.*
☒	☐	For Bayesian analysis, information on the choice of priors and Markov chain Monte Carlo settings
☒	☐	For hierarchical and complex designs, identification of the appropriate level for tests and full reporting of outcomes
☒	☐	Estimates of effect sizes (e.g. Cohen's *d*, Pearson's *r*), indicating how they were calculated

Our web collection on statistics for biologists contains articles on many of the points above.

Software and code

Policy information about availability of computer code

Data collection	Zeiss Zen Blue 2.6, Zeiss Zen Black 2.3 SP1, Nikon NIS-Elements Ar 5.11
Data analysis	Image analysis was performed using Cell Plot v1.3 , ImageJ 1.52p and Chemotaxis and Migration Tool 2.0 (ibidi). Graphpad Prism 8.4.3 was used to analyze statistics. Ilastik 1.3.0 was used for segmentation of EM data. Blender 2.80 was used for rendering and Neuromorph 2.0 toolkit for superimposing rendered with original images.

For manuscripts utilizing custom algorithms or software that are central to the research but not yet described in published literature, software must be made available to editors and reviewers. We strongly encourage code deposition in a community repository (e.g. GitHub). See the Nature Research guidelines for submitting code & software for further information.

Data

Policy information about availability of data

All manuscripts must include a data availability statement. This statement should provide the following information, where applicable:

- Accession codes, unique identifiers, or web links for publicly available datasets
- A list of figures that have associated raw data
- A description of any restrictions on data availability

All FIB-SEM data are deposited to EMPIAR under accession codes EMPIAR-11002 and EMPIAR-11003. Other data supporting the findings of this study are available from the corresponding author on reasonable request.

Field-specific reporting

Please select the one below that is the best fit for your research. If you are not sure, read the appropriate sections before making your selection.

- [x] Life sciences
- [ ] Behavioural & social sciences
- [ ] Ecological, evolutionary & environmental sciences

For a reference copy of the document with all sections, see nature.com/documents/nr-reporting-summary-flat.pdf

Life sciences study design

All studies must disclose on these points even when the disclosure is negative.

Sample size

Sample size was determined according to our own experience using similar experimental designs described in this manuscript (e.g. Gomes et. all Cell 2005, Luxton et al. Science 2010, Calero-Cuenca et al. Curr. Biol. 2021). Sample size of FIB-SEM and TEM was determined by the limited access to the equipment at EMBL EM facility. All experiments were performed at least 3 times independently and sample size was sufficient for most experiments (at least 18 cells). The only exception were the FIB-SEM, and the TEM experiments where only one cell per condition was acquired, in a total of one experiment. Moreover, the experiment of Fig. Sup. 4 was only performed in two independent experiments.

Data exclusions

No data exclusion was performed.

Replication

The experimental findings were reliably reproduced as validated by at least two independent experiments. In vitro experiments were performed in triplicate whenever possible.

Randomization

Cell culture dishes obtained were randomly distributed into experimental groups.

Blinding

No blinding was performed. Blinding was not required to group allocation during data collection and/or analysis since this is the standard approach for the experiments described in the manuscript, as there were no subjective measurements.

Reporting for specific materials, systems and methods

We require information from authors about some types of materials, experimental systems and methods used in many studies. Here, indicate whether each material, system or method listed is relevant to your study. If you are not sure if a list item applies to your research, read the appropriate section before selecting a response.

Materials & experimental systems

n/a	Involved in the study	
[ ]	[x]	Antibodies
[ ]	[x]	Eukaryotic cell lines
[x]	[ ]	Palaeontology and archaeology
[x]	[ ]	Animals and other organisms
[x]	[ ]	Human research participants
[x]	[ ]	Clinical data
[x]	[ ]	Dual use research of concern

Methods

n/a	Involved in the study	
[x]	[ ]	ChIP-seq
[x]	[ ]	Flow cytometry
[x]	[ ]	MRI-based neuroimaging

Antibodies

Antibodies used

The primary antibodies used for immunofluorescence The primary antibodies used for immunofluorescence were: rabbit anti-β-Catenin (712700, 1:200, Invitrogen), mouse anti-Pericentrin (611814, 1:200, BD-Biosciences), rabbit anti-Nesprin-2G (1:200, gift from Gregg Gundersen), rabbit anti-Kinectin-1 (HPA003178, 1:200, Sigma-Aldrich), rabbit anti-p180 (HPA011924, 1:200, Sigma-Aldrich), rat anti-tyrosinated α-tubulin (92092402, 1:50, Clone YL1/2, European Collection of Animal Cell Cultures, Salisbury, UK), chicken anti-GFP (GFP-1020, 1:1000, Aves Labs). The anti-rat (A21247, A21434, A21208) anti-mouse (A21424, A21236, A21202), anti-rabbit (A21429, A21245, A21206) and anti-chicken (A11039, A-11040, A32933) secondary antibodies used were Alexa Fluor 488, Alexa Fluor 555 and Alexa Fluor 647 (1:800, Life Technologies). All of the antibodies and probes that were used are listed in Supplementary Table 2.

The antibodies used for Western Blot were rabbit anti-Climp-63 (HPA041143, 1:500, Sigma-Aldrich), rabbit anti-Vinculin (V9131, 1:500, Sigma-Aldrich), rabbit Myosin-1C (HPA001768, 1:1000, Sigma-Aldrich), rabbit Reticulon-4 (HPA023977, 1:1000, Sigma-Aldrich) and mouse Tubulin (T6557, 1:1000, Sigma-Aldrich). Secondary antibodies used were anti-rabbitHRP (Thermo Scientific #31460, 1:5000) and anti-mouse HRP (Thermo Scientific #32430, 1:5000).

Validation

rabbit anti-β-Catenin (712700, 1:200, Invitrogen), https://www.thermofisher.com/antibody/product/beta-Catenin-Antibody-clone-CAT-15-Polyclonal/71-2700

mouse anti-Pericentrin (611814, 1:200, BD-Biosciences), https://www.bdbiosciences.com/en-us/products/reagents/microscopy-imaging-reagents/immunofluorescence-reagents/purified-mouse-anti-mouse-pericentrin.611814

rabbit anti-Nesprin-2G (1:200, gift from Gregg Gundersen) – Gomes et al Cell 2005.,
rabbit anti-Kinectin-1 (HPA003178, 1:200, Sigma-Aldrich),https://www.sigmaaldrich.com/PT/en/product/sigma/hpa003178

rabbit anti-p180 (HPA011924, 1:200, Sigma-Aldrich), https://www.sigmaaldrich.com/PT/en/product/sigma/hpa011924

rat anti-tyrosinated α-tubulin (92092402, 1:50, Clone YL1/2, European Collection of Animal Cell Cultures, Salisbury, UK) – https://www.culturecollections.org.uk/products/celllines/hybridoma/detail.jsp?refId=92092402&collection=ecacc_hc

chicken anti-GFP (GFP-1020, 1:1000, Aves Labs).- http://www.aveslab.com/products/epitope-tag-and-gfp-antibodies/anti-gfp-green-fluorescent-protein-antibodies-2

anti-Climp-63 (HPA041143, 1:500, Sigma-Aldrich), http://www.sigmaaldrich.com/catalog/product/sigma/hpa041143?lang=pt&region=PT

rabbit anti-Vinculin (V9131, 1:500, Sigma-Aldrich), http://www.sigmaaldrich.com/catalog/product/sigma/v9131?gclid=CjwKEAjwps2_BRC5jduHor-h8xESJADGT-LtFOHgOPhuSAaw3JluARuEKE-BaatYCtTA0Fh5zak52RoCxQvw_wcB&lang=pt&region=PT

rabbit Myosin-1C (HPA001768, 1:1000, Sigma-Aldrich), http://sigmaaldrich.com/catalog/product/sigma/hpa001768?lang=pt&region=PT

rabbit Reticulon-4 (HPA023977, 1:1000, Sigma-Aldrich), https://www.sigmaaldrich.com/catalog/product/sigma/hpa023977?lang=pt&region=PT

mouse Tubulin (T6557, 1:1000, Sigma-Aldrich), http://www.sigmaaldrich.com/catalog/search?N=0&focus=product&interface=All&lang=pt&mode=match+partialmax&region=PT&term=T6557

Eukaryotic cell lines

Policy information about cell lines

Cell line source(s)	U2OS, NIH3T3 and HEK293Tcells were obtained from ATCC
Authentication	Cell lines used were obtained from a comercial provider (ATCC) and thus not subsequently validated.
Mycoplasma contamination	Cell lines tested negative for micoplasma contamination.
Commonly misidentified lines (See ICLAC register)	None were used.
